# Supplementary material for: Compliance of Adolescent Friendly Health Clinics with National and International Standards: Quantitative findings from the i-Saathiya study
Source: BMJ Open. 2024 Feb 13;14(2):e078749. doi: 10.1136/bmjopen-2023-078749 (PMC10868312; doi:10.1136/bmjopen-2023-078749)
Supplement: Supplementary data [file bmjopen-2023-078749supp001.pdf]

Table S1: Compliance of Adolescent Friendly Health Clinics (AFHCs) with RKSK Benchmarks: Item-Wise Scores.

Items Under Each Benchmark	Method of assessment<br>Observation /<br>Observation and verified by interaction	AFHCs with Presence of item<br>N (%)
**Infrastructure- clean, bright and colorful (Max. Marks= 5)**		
Overall infrastructure of AFHC is bright with wall paints, color, furniture	Observation	6 (42.9)
Is the toilet clean?	Observation	5 (35.7)
Are the surroundings of the facility clean?	Observation	11 (78.5)
Does the waiting area have adequate lighting?	Observation	9 (64.3)
Does the waiting area seem clean overall?	Observation	8 (57.1)
**Can be easily accessed by the adolescents (distance and convenient working hours) (Max. Marks= 3)**		
Do the functional days of the AFHC match with the RKSK operational framework?	Observation and verified by interaction	11 (78.5)
Do the Operational Hours of the AFHC match with the RKSK operational framework?	Observation and verified by interaction	11 (78.5)
Is AFHC accessible to adolescents i.e. where adolescents can reach using the local transport?	Observation	14 (100)
**Awareness about the Clinic and its range of services (IEC, proper signage etc.) (Max. Marks= 6)**		
Signboard mentions that it is the AFHC	Observation	10 (71.4)
Signboard that mentions the facility's operating hours?	Observation	8 (57.1)
Signboard clearly visible?	Observation	8 (57.1)
Signboard displayed in the regional language?	Observation	7 (50)
Is the IEC material displayed in regional language?	Observation	5 (35.7)
Does the waiting area have information, education and communication materials specifically developed for adolescents?	Observation	6 (42.9)

**Maintains privacy and confidentiality (Max. Marks= 10)**		
Is the waiting area in a common area ?	Observation	8 (57.1)
AFHC is separated from general OPD	Observation	10 (71.4)
Separate area for Counselling services	Observation	9 (64.3)
Separate area for Clinical services	Observation	4 (28.6)
Separate area for commodities disbursement	Observation	5 (35.7)
There are curtains on the doors and windows	Observation	8 (57.1)
Case records are kept in a secure place, accessible only to authorized personnel	Observation and verified by interaction	13 (92.9)
The registers are kept under lock and key outside operating hours	Observation and verified by interaction	11 (78.5)
Information on the identity of the adolescent and the presenting issue are gathered in confidence during registration	Observation and verified by interaction	9 (64.3)
No one can see or hear an adolescent client from the outside during the consultation or counselling	Observation	9 (64.3)
**Referral from the periphery/community and further referral linkages with the higher facilities and specialty clinics. (Max. Marks= 1)**		
Referral guidelines	Observation and verified by interaction	1 (7.1)
