# Supplementary material for: Compliance of Adolescent Friendly Health Clinics with National and International Standards: Quantitative findings from the i-Saathiya study
Source: BMJ Open. 2024 Feb 13;14(2):e078749. doi: 10.1136/bmjopen-2023-078749 (PMC10868312; doi:10.1136/bmjopen-2023-078749)
Supplement: Supplementary data [file bmjopen-2023-078749supp002.pdf]

Table S2: Compliance of Adolescent Friendly Health Clinics (AFHCs) with WHO Global standards for quality health-care services for adolescents: Item-Wise Scores.

Items Under Each Standard	Method of assessment<br>Observation<br>Observation and verified by interview	Yes
**Standard 1- Adolescents' health literacy (Max. Score= 3)**		
Signboard that mentions the facility's operating hours?	Observation	8 (57.1)
Signboard clearly visible?	Observation	8 (57.1)
Does the waiting area have information, education and communication materials specifically developed for adolescents?	Observation	6 (42.9)
**Standard 3- Appropriate package of services (Max. Score= 2)**		
Guidelines/SOPs for which services should be provided in the facility and in the community	Observation and verified by interview	0 (0)
Referral guidelines	Observation and verified by interview	1 (7.1)
**Standard 4- Providers' competencies (Max. Score= 10)**		
BMI growth charts for adolescents	Observation	3 (21.5)
The rights of adolescents to information, non-judgmental attitude and respectful care	Observation and verified by interview	1 (7.1)
The policy commitment of the health facility to provide health services to all adolescents without discrimination	Observation and verified by interview	1 (7.1)
The policy on confidentiality and privacy	Observation and verified by interview	0 (0)
The policy and procedure to ensure free or affordable service provision for adolescents	Observation and verified by interview	0 (0)
Job Description- Medical Officer	Observation and verified by interview	1 (7.1)

Job Description- Staff Nurse	Observation and verified by interview	0 (0)
Job Description- ANM	Observation and verified by interview	0 (0)
Job Description- Counsellor	Observation and verified by interview	1 (7.1)
Job Description- Specialist	Observation and verified by interview	0 (0)
**Standard 5- Facility characteristics (Max. Score= 34)**		
Does the waiting area have information, education and communication materials specifically developed for adolescents?	Observation	6 (42.9)
Does the waiting area have drinking water?	Observation	0 (0)
Does the waiting area seem welcoming overall?	Observation	6 (42.9)
Does the waiting area seem clean overall?	Observation	8 (57.1)
Is the toilet clean?	Observation	5 (35.7)
Does the facility have permanent electricity during working hours?	Observation	9 (64.3)
Does the facility have safe waste disposal?	Observation	3 (21.5)
Does the facility have safe storage and disposal of sharps?	Observation	3 (21.5)
Does the facility have adequate hand hygiene facilities that are located in or adjacent to the office/examination room?	Observation	6 (42.9)
Does the toilet have functioning hand hygiene facilities?	Observation	6 (42.9)
Are the surroundings of the facility clean?	Observation	11 (78.5)
Blood pressure measurement machine	Observation	2 (14.3)
Stethoscope	Observation	3 (21.5)

Clinical thermometer	Observation	2 (14.3)
Weighing scale	Observation	10 (71.4)
Measuring tape	Observation	1 (7.1)
Height Meter/Stadiometer	Observation	2 (14.3)
Hemoglobin Testing	Observation	3 (21.5)
Provision of Condoms	Observation and verified by interview	6 (42.9)
Provision of Oral Contraceptive Pills	Observation and verified by interview	3 (21.5)
Provision of Emergency Contraceptive Pills	Observation and verified by interview	1 (7.1)
Provision of Pregnancy Testing Kits	Observation and verified by interview	2 (14.3)
Paracetamol	Observation and verified by interview	3 (21.5)
Guidelines/SOPs on protecting the privacy and confidentiality of adolescents	Observation and verified by interview	0 (0)
Guidelines/SOPs on including staff responsibilities for making the health facility welcoming, convenient and clean	Observation and verified by interview	0 (0)
SOPs on how to minimize waiting time	Observation and verified by interview	0 (0)
SOPs on how to provide services to adolescents with or without an appointment	Observation and verified by interview	0 (0)
There are curtains on the doors and windows	Observation	8 (57.1)
Communication between reception staff and visitors is private and cannot be overheard, including from the waiting room	Observation	2 (14.3)
Case records are kept in a secure place, accessible only to authorized personnel	Observation and verified by interview	13 (92.9)
The registers are kept under lock and key outside operating hours	Observation and verified by interview	11 (78.5)

Information on the identity of the adolescent and the presenting issue are gathered in confidence during registration	Observation	9 (64.3)
In the offices/examining rooms, there is a screen to separate the examination area from the consultation area	Observation	4 (28.6)
No one can see or hear an adolescent client from the outside during the consultation or counselling	Observation	9 (64.3)
**Standard 6- Equity and non-discrimination (Max. Score= 4)**		
The policy commitment of the health facility to provide health services to all adolescents without discrimination	Observation and verified by interview	1 (7.1)
The policy and procedure to ensure free or affordable service provision for adolescents	Observation and verified by interview	0 (0)
Guidelines/SOPs on applying policies for free, or affordable, service provision to adolescents	Observation and verified by interview	0 (0)
Guidelines/SOPs on equitable service provision to all adolescents irrespective of their ability to pay, age, sex, marital status or other characteristics	Observation and verified by interview	0 (0)
**Standard 7- Data and quality improvement (Max. Score= 3)**		
Enrolment Register	Observation and verified by interview	14 (100)
Counselling Register	Observation and verified by interview	12 (85.7)
Stock Register	Observation and verified by interview	2 (14.3)
**Standard 8- Adolescents' participation (Max. Score= 1)**		
Guidelines/SOPs on informed consent	Observation and verified by interview	0 (0)
