# Supplementary material for: Compliance of Adolescent Friendly Health Clinics with National and International Standards: Quantitative findings from the i-Saathiya study
Source: BMJ Open. 2024 Feb 13;14(2):e078749. doi: 10.1136/bmjopen-2023-078749 (PMC10868312; doi:10.1136/bmjopen-2023-078749)
Supplement: Author's manuscript [file bmjopen-2023-078749.draft_revisions.pdf]

BMJ Open is committed to open peer review. As part of this commitment we make the peer review history of every article we publish publicly available.

When an article is published we post the peer reviewers' comments and the authors' responses online. We also post the versions of the paper that were used during peer review. These are the versions that the peer review comments apply to.

The versions of the paper that follow are the versions that were submitted during the peer review process. They are not the versions of record or the final published versions. They should not be cited or distributed as the published version of this manuscript.

BMJ Open is an open access journal and the full, final, typeset and author-corrected version of record of the manuscript is available on our site with no access controls, subscription charges or pay-per-view fees (http://bmjopen.bmj.com).

If you have any questions on BMJ Open's open peer review process please email info.bmjopen@bmj.com

BMJ Open

Compliance of Adolescent-Friendly Health Clinics with National and International Standards: Qualitative Findings from the i-Saathiya Study

Journal:	*BMJ Open*
Manuscript ID	bmjopen-2023-078749
Article Type:	Original research
Date Submitted by the Author:	10-Aug-2023
Complete List of Authors:	Bahl, Deepika; Public Health Foundation of India, Health Promotion Division<br>Bassi, Shalini; Public Health Foundation of India, Health Promotion Division<br>Maity, Heeya; Public Health Foundation of India<br>Krishnan, Supriya ; Public Health Foundation of India<br>Dringus, Stefanie; independent global health consultant<br>Mason-Jones, Amanda; University of York, Health Sciences<br>Malik, Anku; Public Health Foundation of India<br>Arora, Monika; Public Health Foundation of India, Health Promotion Division
Keywords:	Adolescent, Health Services Accessibility, PUBLIC HEALTH

SCHOLARONE™
Manuscripts

I, the Submitting Author has the right to grant and does grant on behalf of all authors of the Work (as defined in the below author licence), an exclusive licence and/or a non-exclusive licence for contributions from authors who are: i) UK Crown employees; ii) where BMJ has agreed a CC-BY licence shall apply, and/or iii) in accordance with the terms applicable for US Federal Government officers or employees acting as part of their official duties; on a worldwide, perpetual, irrevocable, royalty-free basis to BMJ Publishing Group Ltd ("BMJ") its licensees and where the relevant Journal is co-owned by BMJ to the co-owners of the Journal, to publish the Work in this journal and any other BMJ products and to exploit all rights, as set out in our licence.

The Submitting Author accepts and understands that any supply made under these terms is made by BMJ to the Submitting Author unless you are acting as an employee on behalf of your employer or a postgraduate student of an affiliated institution which is paying any applicable article publishing charge ("APC") for Open Access articles. Where the Submitting Author wishes to make the Work available on an Open Access basis (and intends to pay the relevant APC), the terms of reuse of such Open Access shall be governed by a Creative Commons licence – details of these licences and which Creative Commons licence will apply to this Work are set out in our licence referred to above.

Other than as permitted in any relevant BMJ Author's Self Archiving Policies, I confirm this Work has not been accepted for publication elsewhere, is not being considered for publication elsewhere and does not duplicate material already published. I confirm all authors consent to publication of this Work and authorise the granting of this licence.

For peer review only

Title: Compliance of Adolescent-Friendly Health Clinics with National and International Standards: Qualitative Findings from the i-Saathiya Study

Authors: Deepika Bahl[1], Shalini Bassi[1], Heeya Maity[1], Supriya Krishnan[1], Stefanie Dringus[2], Amanda J. Mason-Jones[3], Anku Malik[1], Monika Arora[1]

Affiliations

[1]Health Promotion Division, Public Health Foundation of India, Delhi, India.

[2]Independent Global Health Consultant, London, England.

[3]Department of Health Sciences, University of New York, Heslington, York, England.

For peer review only

For peer review only - http://bmjopen.bmj.com/site/about/guidelines.xhtml

Abstract

Objective: Indian adolescents experience several health challenges requiring acceptable, equitable, appropriate, and effective healthcare services. Our objective was to assess the compliance of Adolescent Friendly Health Clinics (AFHCs) in two of India's largest states, using both national benchmarks (under Rashtriya Kishor Swasthya Karykaram-RKSK) and global standards (World Health Organisation standards).

Design: Cross-sectional study comprising semi-structured observations and interviews was conducted (November 2021 to June 2022).

Setting: Fourteen Adolescent Friendly Health Clinics across all health system levels were included from two districts of Maharashtra (n=8) and Madhya Pradesh (n=6). These AFHCs were observed, and at few places observations were verified with interviews of health workers (Medical Officer/Auxillary Nurse Midwife/ Counsellors) handlings adolescents. The developed checklist included 57 items based on adapted global standards and national benchmarks.

Result: High compliance of AFHCs with RKSK benchmarks was attributed to various items including the accessibility of AFHCs through local transport (n=14, 100%), clean surroundings (n=11, 78.5%), presence of signage (n=10, 71.4%), convenient operating days and time (n=11, 78.5%), and secure storage of records (n=13, 92.9%). Concurrently, items in AFHCs which demanded attention encompassed, the availability of Information, Education and Communication resources, which were deficient in 57.1% of AFHCs (n=8). Similarly, designated areas for clinical services (n=10, 71.4%) and commodity disbursement (n=9, 64.3%) lacked in more than half of recruited AFHCs. Additionally, lack of guidelines for referrals (n=13, 92.9%), as well as Standard Operating Procedures designed to ensure equity,

For peer review only - http://bmjopen.bmj.com/site/about/guidelines.xhtml

For peer review only

non-judgemental attitude, competence, confidentiality and referral as per World Health Organisation standards.

Conclusion: Evidence spotlights the strengths and gaps in AFHCs, aligning with, government's priorities on adolescent health. Addressing the identified gaps is crucial to create healthcare facilities that are adolescent-friendly, easily accessible, and effectively navigate adolescent challenges. This concerted effort would contribute to their development and transformation, playing a pivotal role in India's progress.

Strengths and limitations of the study

- The study provides insights into the existing quality of adolescent-friendly healthcare services in India and pathways for strengthening to meet global standards.
- Given that the data was gathered from two states only, the applicability of the findings may not be generalizable to the entire country.
- Inclusion of exit interviews with adolescent clients to understand their perceptions would have allowed for a more comprehensive evaluation of the services.

Introduction

Adolescent-Friendly Health Services (AFHS) are evidence-based interventions to address health concerns by offering accessible, acceptable, equitable, and appropriate[1] healthcare services for adolescents. In the South-East Asian Region, countries are prioritizing adolescent health and well-being by scaling AFHS through their national public health systems. Multiple delivery settings and approaches including clinics, school health programs, community outreach, partnerships with civil society, development partners and professional bodies are utilised[2] to provide these services. Evidence highlights that these adolescent-friendly services are cost-effective in delivering sexual and reproductive health (SHR) services[3] to adolescents.

For peer review only - http://bmjopen.bmj.com/site/about/guidelines.xhtml

For peer review only

In India, AFHS is provided through Adolescent Friendly Health Clinics (AFHCs) as part of 'Rashtriya Kishor Swasthya Karyakram-RKSK' (India's Adolescent Health Strategy) launched in 2014[4]. AFHC is one of the critical pillars of the RKSK that seek to enable all adolescents to realize their full potential by making informed decisions concerning their health, and by accessing the services and support to implement their decisions. These clinics provide clinical and counseling services related to nutrition, injuries, non-communicable diseases, mental health, substance misuse and sexual and reproductive health to adolescents[5]. AFHCs have been integrated into medical colleges, district and sub-district hospitals, community health centres (CHCs)/ rural hospitals, and primary health centres (PHCs)[6] to provide services for adolescents. Despite years of establishment, the available evidence from India highlights the low footfall of adolescents in AFHCs[7-9]. This is partly explained by multiple barriers in utilizing these services including a lack of adolescents' knowledge about the AFHCs in their vicinity[10], inadequate staffing, untrained service providers, social taboo, inadequate equipment, low priority accorded by Program Managers, and adolescents perceive that services are restricted to Reproductive and Sexual Health [11]. Thus,resulting in a missed opportunity in identifying and treating health problems early and assisting adolescents in developing positive healthcare-seeking behaviors[12].Thus, undermines the goal of RKSK to promote adolescents' health and well-being and emphasizes the need for a standardized approach to enhance the quality of care[13] across India for effective utilization by adolescents.

With this,as part of the i-Saathiya study, which aims to explore the implementation of the peer education program, we also assessed AFHC compliance with both national[14] and global standards for quality healthcare services catering to adolescents [13.] This assessment was carried out in the states of Madhya Pradesh and Maharashtra (India) through semi-structured observations and interview-based varifications. This method was suitable to achieve this

For peer review only - http://bmjopen.bmj.com/site/about/guidelines.xhtml

objective as it allowed us to see all activities directly, rather than solely relying on subjective opinions and perceptions[15]. The findings of this study will be useful in strengthening the Indian AFHCs, enabling them to safeguard, promote and improve adolescent health by providing acceptable, equitable, appropriate, and effective healthcare services; and establishing safe and supportive environments to facilitate their growth and development[16].

Methodology

Study Setting and Sample Selection

AFHCs in two districts of Madhya Pradesh (Panna and Damoh) and Maharashtra (Yavatmal and Nashik) were observed. Panna and Damoh are situated in Madhya Pradesh, a large state in central India with a total population of 72.7 million[17] and a literacy rate of 69.3%. Yavatmal and Nashik are in Maharashtra, which is situated in the Western Peninsular region of India with an approximate population of 112.4 million[17] and an 82.3% literacy rate. The criteria for selection of states and districts and have been described in another i-Saathiya publication[18]. Table 1 lists the AFHCs at various levels of the health system in selected study districts and blocks of both states. To assess compliance, we included one AFHC at the district-level (District Hospital -DH) or Government Medical College (GMC), in all four selected study districts (n=4). Each district of Maharashtra featured one AFHC at the Sub-district Hospital (SDH, n=2) Considering a higher number of AFHCs at the lower level of the health system, we adopted a random selection approach to choose one Community Health Center (CHC) or Rural Hospital (RH) (n=4) and Primary Health Center (PHC) from each study district (n=4), using a fishbowl method. In total, 14 AFHCs were included using this methodology.

Table 1: Number[#] of AFHCs at various levels of health system in the study districts and blocks

State	District	Blocks	Level of Health Facility

For peer review only - http://bmjopen.bmj.com/site/about/guidelines.xhtml

			**Government Medical College**	**District Hospital**	**Sub District Hospital***	**CHCs/RH under each block**	**PHCs under each block**
Madhya Pradesh	Panna	Panna	No	1	No	1	4
		Ajaygarh				1	4
		Shahnagar				1	2
		Pawai				1	5
	Damoh	Damoh	No	1	No	1	2
		Jabera				1	2
		Patera				1	2
		Patharia				1	2
Maharashtra	Yavatmal	Babulgaon	1	No	No	1	2
		Pusad			1	No	2
		Yavatmal			No	No	2
		Zari-Jamani			No	1**	2
	Nashik	Dindori	No	1		2** (RH equivalent to CHC)	2
		Nandgaon			1	1**	2
		Sinner				1**	2
		Surgana				2**	2
Total			1	3	2	16	39

#AFHC numbers during November 2021- June 2022

* SDH only exist in Maharashtra

** RH equivalent to CHC

For peer review only

Study design

A cross-sectional study using qualitative methods was conducted between November 2021 and June 2022.

Data collection

The trained study team conducted semi-structured observations using a printed structured checklist that included several performance criteria. this assessment was carried out in fourteen clinics, out of sixty-one clinics in the selected districts to assess the compliance of AFHCs. The checklist was developed by adapting the World Health Organization's (WHO) Global standards for quality healthcare services for adolescents to align with the Indian context[13,14] and using RKSK benchmarks[14]. The adaptation was carried through a Delphi process[20] involving panel of four adolescent health experts. Throughout the process, several items were excluded from the checklist, such as vaccine availability, ophthalmoscope, injectable contraceptives, refrigerator, contraceptive implants, etc. These exclusions were guided by the Adolescent Health Strategy (2014), which outlines the essential requisites for AFHCs[6]. All trained staff also completed the Good Clinical Practice course offered by National Drug Abuse Treatment Clinical Trials Network (NDAT CTN).

The developed checklist comprised a total of 82 items (WHO standards=57 items; RKSK Benchmarks=25 items). Some of the items like Standard Operating Procedures, policy commitments, availability of condoms, contraceptives, etc. were observed and their responses were further confirmed through interviews conducted with the staff members [counsellors/ Auxillary Nurse Midwives (ANMs) / Medical Officer (MOs)] on the same day and time. The embedded interview approach was necessary as specific items could be securely stored and

For peer review only - http://bmjopen.bmj.com/site/about/guidelines.xhtml

concealed from direct observation. Table S1 provides the list of items verified by the interview with the above-mentioned staff due to their involvement in handling adolescents and their health issues within AFHCs. The printed checklist was pre-tested by the study team in one AFHC within each study state, to assess its feasibility in the Indian context. Feedback received from the pre-testing primarily focused on the sequence of items, including potential deletion or addition. These inputs were incorporated into the final version of the checklist used for the main study. The two facilities used for the pre-testing phase were not included in the main study.

The observations were scheduled in consultation with the concerned authorities (counselors/ ANMs/ MOs) at the facility, who were informed of the objective of conducting these assessments. Written informed consent was sought from the concerned staff of the health care facility before commencing the data collection. To avoid any potential bias in the responses, the checklist was not shared with the AFHC staff before data collection. The data collection process in each facility persisted approximately 90-120 minutes.

The ethical clearance for the study was obtained from the Institutional Ethics Committee of the Public Health Foundation of India (Reference # TRC-IEC-342.1/17) along with approvals from the Indian Health Ministry's Screening Committee (2017-2250).

Measures

The RKSK operational framework[6,14] outlines seven benchmarks designed to ensure the provision of 'friendly' facility-based clinical and counseling services for adolescents at AFHC. Out of the seven benchmarks, only five benchmarks listed in Table 2 have been assessed in this study. The assessment of two benchmarks i.e. non-judgmental and competent health service providers, and awareness of the services to the community members was not feasible. Evaluating these benchmarks would have required exit interviews and observations of

For peer review only

interaction between counsellors and adolescents, as well as interviews with community members. Unfortunately, these aspects fell beyond the scope of our study. The checklist included for assessing the RKSK benchmarks consisted of 25 items, detailed in Table 2, and Table S1.

Table 2. Variables assessing RKSK Benchmarks and International Standards

**RKSK Benchmarks[6,14]**	**Number of items included***	**Objective**
Infrastructure- clean, bright and colourful	5	Provision of attractive, clean and comfortable health care facility to avail services.
Accessibility by adolescents (distance and convenient working hours)	3	Ensures easy access to health care facility through convenient working days/hours and local transportation.
Awareness about the clinic and range of service it provides (IEC, Proper Signage)	6	Provision of clearly visible sign board in regional language and information, education, and communication material to provide awareness about the presence of health facility and services available
Privacy and confidentiality	10	Ensures privacy in service provision through segregated areas for different services and maintain confidentiality by securing case records and registers under lock and key, accessible to only authorised AFHC staff
Referral from the periphery/community and further referral linkages with the higher facilities and specialty clinics	1	Provision of referral services to higher facilities to improve quality of healthcare among adolescents.
**Total Items**	25	
**WHO global standards for quality health care services for adolescents[13]**		
Standard 1- Adolescents' health literacy	3	To make adolescents knowledgeable about their own health and aware about where and when to receive health services.
Standard 2- Community Support**	0	To ensure that parents, guardians, other community members and community organizations recognize the value of providing

For peer review only - http://bmjopen.bmj.com/site/about/guidelines.xhtml

		health services to adolescents and support all adolescents to utilise the health services they need
Standard 3- Appropriate package of services	2	Provision of package of services including information, counselling, diagnostic, treatment and care at the health facility to fulfil the needs of all adolescents.
Standard 4- Providers' competencies	10	Healthcare providers' attitudes, knowledge and skills are at the core of quality service provision. This standard ensures that health-care providers have the technical competence required to provide effective health services to adolescents. Adolescents' rights to information, privacy, confidentiality, non-discrimination, non-judgemental attitude and respect are protected and fulfilled by the healthcare providers
Standard 5- Facility characteristics	34	The health facility has convenient operating hours, a welcoming and clean environment and maintains privacy and confidentiality. It has the equipment, medicines, supplies and technology needed to ensure effective service provision to adolescents.
Standard 6- Equity and non-discrimination	4	Ensures provision of equitable care to all adolescents irrespective of their ability to pay, age, sex, marital status, education, ethnic origin, sexual orientation or other characteristics.
Standard 7- Data and quality improvement	3	The standard, stresses on the importance of the facility's actions to collect, analyse and use data on cause-specific service utilization and quality of care, disaggregated by age and sex to support quality improvement
Standard 8- Adolescents' participation	1	Ensures adolescents' participation in the planning, monitoring and evaluation of health services and in decisions regarding their own care. It also emphasizes on adolescents' participation in certain aspects of service provision.

For peer review only - http://bmjopen.bmj.com/site/about/guidelines.xhtml

Total	57	

*Details of the items are present in Table S1

** Standard 2 not assessed since exit interviews with adolescents were not conducted.

Similarly, to assess compliance with international standards, seven out of eight global standards defined in the WHO global standards for quality healthcare services for adolescents[13] were included in the checklist (Table 2). Community support (Standard 2) was not assessed as exit interviews with adolescent clients were needed. The developed checklist (Table 2 and S2) included only 57 items covering the WHO standards relevant to the Indian context [21].

Data analysis

Data were entered into EpiInfo[22] and extracted into Excel for coding, cleaning, and analysis. For assessing compliance with international standards, a score was created for each standard as described in the WHO scoring guidelines[23]. Each item in the checklist was dichotomous (yes/no) with "Yes" awarded 1 point, and "No" awarded 0 point. A summation of all the questions in each standard was used to obtain the total score for that standard. The maximum score was obtained by adding the number of items in that standard. The percentage score (%) for each standard was calculated by taking the score, scored by the AFHC, dividing it by the maximum score for the standard, and then multiplying by 100 for the standard. Percentage scores (%) were classified as: 1) ≤10%: not meeting standard, 2) 10-40%: needs major improvement, 3) 40-80%: needs some improvement, and 4) ≥80% or more: meets the standard[21]. Since, no scoring has been developed for assessing compliance to the RKSK benchmarks, WHO scoring guidelines[23] were adopted for assessing compliance of AFHCs with RKSK benchmarks.

Results

For peer review only - http://bmjopen.bmj.com/site/about/guidelines.xhtml

Out of the total fourteen clinics included, eight were from Maharashtra (Nashik: 4 and Yavatmal: 4), while six were from Madhya Pradesh (Panna: 3 and Damoh: 3). Additionally, two clinics from Maharashtra, due to the presence of an additional level of the health system i.e. sub-district hospital. Table 3 describes the characteristics of the 14 clinics including functional AFHC days and their operating hours. The maximum duration during which adolescents could avail services in a day was seven hours, while the minimum duration was four hours.

Table 3: Descriptive characteristics of AFHCs (n=14)

Clinic ID	AFHC Location at Health System	State	Number of operational days in week	Total Operational Hours
1	Government Medical College	Maharashtra	2	6
2	District Hospital	Maharashtra	5	7
3	District Hospital	Madhya Pradesh	6	7
4	District Hospital	Madhya Pradesh	6	7
5	Sub District Hospital	Maharashtra	6	7
6	Sub District Hospital	Maharashtra	6	4
7	Rural Hospital	Maharashtra	5	4
8	Rural Hospital	Maharashtra	6	2
9	Community Health Centre	Madhya Pradesh	6	7
10	Community Health Centre	Madhya Pradesh	6	7
11	Primary Health Centre	Maharashtra	1	4
12	Primary Health Centre	Maharashtra	1	4
13	Primary Health Centre	Madhya Pradesh	1	4
14	Primary Health Centre	Madhya Pradesh	1	4

For peer review only - http://bmjopen.bmj.com/site/about/guidelines.xhtml

The following section elucidates the compliance of the AFHCs where the assessment was undertaken with national RKSK benchmarks and WHO global standards of quality health services for adolescents.

RKSK Benchmarks

Infrastructure- clean, bright and colourful

Out of 14 AFHCs, half of the AFHCs required only some improvement (percentage score 40%-80%) in providing clean, bright and colorful infrastructure, while 5 AFHCs (35.7%) met this benchmark as their score was ≥80%. One AFHC (7.1%) required major improvements as the score was between 10%-40% and the other (7.1%) lacked compliance as the percentage score was 0 (Table 4). The item-wise analysis (Table S1) spotlights that as per the observations, the majority of the clinics (n=11, 78.5%) had clean surroundings and more than half had adequate lighting in the waiting area (n=9, 64.3%), and clean waiting areas (n=8, 57.1%). A significant attention is required regarding toilet cleanliness (n=9, 64.3%) and the wall paints, furniture (n=8, 57.1%).

Accessibility by the adolescents (distance and convenient working hours)

Table 4 showed majority of the AFHCs (n=10, 71.4%) had a percentage score ≥80% thereby meeting the benchmark of being accessible concerning distance and working hours to adolescents. As observed, all the 14 AFHCs were located at a place that was easily accessible via local transport such as buses and auto-rickshaws. Based on observations and interviews with AFHC staff, it showed that in the majority of AFHCs (n=11) the timing and functional days were as per the RKSK operational guidelines (Table S1). None of the AFHCs showed non-compliance (percentage score ≤10%) in terms of accessibility (Table 4).

Awareness about the clinic and range of service it provides (IEC, Proper Signage)

For peer review only - http://bmjopen.bmj.com/site/about/guidelines.xhtml

Regarding generating awareness about the clinic using signages and the IEC materials, it was seen that three AFHCs (21.3%) achieved a perfect score of 100% while only one AFHC failed to meet the benchmark, with a percentage score below than 10%. Of the remaining AFHCs, five (35.7%) needed major improvement as the percentage score was between 10%-40%, and the other five AFHCs (35.7%) required some improvement, having a percentage score between 40%-80% (Table 4). Item-wise analysis (Table S1) revealed that a significant number of AFHCs (n=10; 71.4%) had external signboards designating them as AFHC. Visibility and inclusion of operating hours were observed in eight clinics (57.1%). The item which lacked in more than half of the AFHCs was the availability of IEC material in regional language (n=9, 64.3%) and education material in the waiting area (n=8; 57.1%)

Privacy and confidentiality

Overall, the majority of the AFHCs (n=13, 92.9%) required only some improvements in maintaining the privacy and confidentiality of adolescents as the percentage score was between 40%-80%. Only a single AFHC managed to fulfill this benchmark, achieving a percentage score ≥80% (Table 4). The item-wise analysis (Table S1) showed that the majority of clinics (n=10, 71.4%) were physically separated from the general OPD within the healthcare facility. Almost all AFHCs (n=13, 92.9%) maintained adolescent case records in a secure place, accessible only to authorized personnel i.e. counselor or medical officer. This was observed and verified through interviews. Additionally, in 78.5% of AFHCs (n=11), these record registers were kept under lock and key either by the counselor or medical officer..

Observations corroborated by interviews also revealed that during the registration process, the confidentiality of adolescents' identity was maintained. Additionally, no one could see the adolescent client at the time of counseling due to a separate designated area (n=9, 64.3%). However, an area of concern pertains to the provision of separate areas for clinical services

For peer review only - http://bmjopen.bmj.com/site/about/guidelines.xhtml

Table 4: Performance of AFHC as per RKSK Benchmarks				
	Score Percentage			
**RKSK Benchmarks**	**≤10% (not meeting the benchmark)**	**10%-40% (needs major improvement)**	**40%-80% (need some improvement)**	**≥80% (met the benchmark)**
	**N(%)**			
Infrastructure- clean, bright and colorful (Max. Score 5)	1 (7.1)	1 (7.1)	7 (50)	5 (35.7)
Accessibility by the adolescents (distance and convenient working hours) (Max. Score 3)	0 (0)	2 (14.3)	2 (14.3)	10 (71.4)
Awareness about the clinic and range of service it provides (IEC, Proper Signage) (Max. Score 6)	1 (7.1)	5 (35.7)	5 (35.7)	3 (21.3)
Privacy and confidentiality (Max. Score 10)	0 (0)	0 (0)	13 (92.9)	1 (7.1)
Referral from the periphery/community and further referral linkages with the higher facilities and specialty clinics	13 (92.9)	0 (0)	0 (0)	1 (7.1)

(n=10, 71.4%) and the disbursement of commodities (n=9; 64.3%) within the AFHCs (Table S1).

Referral from the periphery/community and further referral linkages with the higher facilities and specialty clinics

Only one AFHC attained a perfect percentage score of 100% signifying the successful accomplishment of the benchmark. While, the remaining AFHCs (92.9%) did not meet the benchmark, as ascertained through observations and verified by interviews. The item-wise analysis showed that only one AFHC had the necessary referral guidelines visibly displayed.

For peer review only

For peer review only - http://bmjopen.bmj.com/site/about/guidelines.xhtml

(Max. score 1)				
N: Number of AFHC<br>% : Percentage of AFHC				

For peer review only

For peer review only - http://bmjopen.bmj.com/site/about/guidelines.xhtml

WHO global standard of quality health services for adolescents

Standard 1: Adolescents' health literacy

Only three AFHCs (21.4%) met this standard with 100% compliance (Table 5), Conversely, an equal number of AFHCs (n=3, 21.4%) lacked compliance, achieving a percentage score lower than 10%. The remaining AFHCs required major (n=3, 21.4%) or some modifications (n= 5, 35.7%) was evident, as their score fall between 10%-40% and 40%-80%, respectively (Table 5). The item-wise analysis highlighted in Table S2 that while a majority of AFHCs (N=8; 57.1%) displayed visible signboards indicating their operating hours, more than half of AFHCs (n=8; 57.1%) lacked resources specifically targeting adolescents for information, education and communication in their waiting areas.

Standard 3: Appropriate package of services

Only one AFHC (7.1%) achieved a percentage score of 50%, indicating the need for some improvement due to the score falling between 40%-80% (Table 5). Conversely, the remaining AFHCs (n=13. 92.9%) fell short of meeting the standards, given their percentage scores were less than 10%. The item-wise analysis (Table S2) highlighted that both reference guidelines (n=13, 92.9) and Standard Operating Procedures (SOPs) (n=14, 100%) for which services to be offered within the facility and community, were not visibly displayed. This was corroborated through interviews.

Standard 4: Providers' competencies

Four AFHCs (28.6%) achieved percentage scores ranged from 10% to 40%, while the remaining ten AFHCs (71.4%) fell short of meeting the standard, receiving percentage scores below 10% (Table 5). The item-wise analysis (Table S2) revealed that low scoring by AFHCs could be attributed to various factors. The absence of well defined job descriptions for health

For peer review only - http://bmjopen.bmj.com/site/about/guidelines.xhtml

care providers such as counselors (n=13, 92.9%), ANMs (n=14, 100%), specialists (n=14, 100%), nurses (n=14, 100%) and medical officers (n=13, 92.9%) can play a role. In addition, the lack of policies addressing aspects like confidentiality and privacy (n=14, 100%), the provision of free or affordable services in all AFHCs (n=14, 100%) and a policy commitment to provide health service to all adolescents without discrimination (n=13, 92.9%) can also be contributing factor to the low scores. The availability of BMI growth charts for adolescents as a supportive tool for providing quality clinical services to adolescents was noted in only three AFHCs (21.5%).

Standard 5: Facility characteristics

Out of the 14 AFHCs evaluated, two (14.3%) demonstrated a need for some improvement, as their percentage scores ranged from 40% and 80%. The remaining AFHCs (n=12, 85.7%) necessitated significant improvements, given their percentage scores were between 10%-40% in order to meet the established standard for facility characteristics (Table 5). Item-wise analysis (Table S2) spotlighted the reasons for the lack of compliance. These included lack of drinking water facility in the waiting area, the non-display of SOPs/ guidelines pertaining to the privacy and confidentiality of adolescents, SOPs outlining staff responsibilities, SOPs for minimizing waiting times, SOPs on how to provide services to adolescents, with or without an appointment. Several other concerns noted in more than 80% of AFHCs were the absence of provision for safe waste disposal, disposal of sharp objects, and the availability of essential equipment like measuring tape, stadiometer, contraceptive pills (oral and emergency), pregnancy kits and blood pressure measurement instruments etc.

Standard 6: Equity and non-discrimination

The majority of the AFHCs (n=13, 92.9%) failed to meet the established standard for equity and non-discrimination (Table 5). Only one AFHC exhibited a limited degree of compliance,

For peer review only - http://bmjopen.bmj.com/site/about/guidelines.xhtml

attaining a percentage score of 25%, which falls within the range of 10%-40%. Table S2 showed that a significant number of AFHCs(n=13;92.9%) did not display a policy commitment to provide health services to all adolescents without any discrimination. Furthermore, other policies, guidelines and procedures related to the provision of free and affordable services, along with equitable services irrespective of sex, marital status, and age were lacking across all clinics, as both observed and verified through interviews (Table S2).

Standard 7: Data and quality improvement

The majority of the AFHCs (n=10, 71.4%) exhibited a need for improvement, as their percentage scores ranged between 40%-80% while two AFHCs (14.3%) achieved a perfect 100% score (Table 5). Table S2, a detailed item-wise analysis showed that all the observed AFHCs (n=14) maintained an enrolment register, and a majority of AFHCs (n=12, 85.7%) possessed counseling registers. However, the stock register was not kept separate for AFHC (n=12, 85.7%)

Standard 8: Adolescents' participation

The assessment of adolescent participation involved inspection of the display of informed consent guidelines or SOPs at the facilities. Unfortunately, none of the fourteen observed AFHCs had any such guidelines or SOPs exhibited, resulting in their failure to adhere to the l WHO standard for quality healthcare services for adolescents (Table 5).

Table 5: AFHC Performance as per the adapted WHO global standards for quality health care services for adolescents[13]

WHO standards	Percentage Score N (%)			
	≤ 10% (not meeting the standard)	10-40% (needs major improvement)	40-80% (needs some improvement)	≥ 80% (met the standard)
**Standard 1- Adolescents' health**	3 (21.4)	3 (21.4)	5 (35.7)	3 (21.4)

For peer review only - http://bmjopen.bmj.com/site/about/guidelines.xhtml

**literacy** (Max. Score 3)				
**Standard 3- Appropriate package of services** (Max. Score 2)	13 (92.9)	0 (0)	1 (7.1)	0 (0)
**Standard 4- Providers' competencies** (Max Score 10)	10 (71.4)	4 (28.6)	0 (0)	0 (0)
**Standard 5- Facility characteristics** (Max. Score 34)	0 (0)	12 (85.7)	2 (14.3)	0 (0)
**Standard 6- Equity and non-discrimination** (Max. Score 4)	13 (92.9)	1 (7.1)	0 (0)	0 (0)
**Standard 7- Data and quality improvement** (Max Score 3)	0 (0)	2 (14.3)	10 (71.4)	2 (14.3)
**Standard 8- Adolescents' participation** (Max Score 1)	14 (100)	0 (0)	0 (0)	0 (0)

Discussion

To the best of our knowledge, this is the first study conducted in India that examines the compliance of AFHCs with both national benchmarks and global standards for quality care. Evidence from previous studies conducted in India have primarily evaluated AFHC compliance based on WHO standards, Indian Public Health Standards, or checklists from the National Health Mission[24-27]. However, adopting a global standard approach further enables critical evaluation of various facets of the quality of AFHS offered at a global level. It would not only

For peer review only - http://bmjopen.bmj.com/site/about/guidelines.xhtml

help minimize variations in service quality but also assist in comparing results across different countries.

RKSK Benchmarks

Our study revealed that most of the AFHCs were compliant with the national benchmarks, indicating the government's commitment to holistic adolescent health, by setting up AFHCs under RKSK. Successful compliance with RKSK benchmarks was indicated by a host of reasons, including awareness of the AFHC through signage, convenient working hours, functional days and clean surroundings. Similar findings have been observed in the state of Ahmedabad [25] and West Bengal [28]. Moreover, AFHCs in both the study states were accessible by local transport, unlike AFHCs in Rajasthan, where ease of accessibility was a major concern[29]. Study results showed that the privacy and confidentiality of the patient were maintained, as all case records were kept in a secured place and only authorized personnel (counsellor, ANM and medical officer) had access to retrieve them. Other requisites pertaining to privacy and confidentiality in adolescent services, like 'the provision of dedicated space for adolescents in clinics' have been one of the pressing issues highlighted in the literature[30-31]. Similar to AFHCs in Rajasthan, Jharkhand and Maharashtra[32], more than half of the AFHCs included in our study, struggled to provide a separate waiting room and a separate area for clinical services and commodity disbursement for adolescents, while contradicting other regional AFHCs in Chandigarh and Kolkata[33]. The lack of these separate areas can threaten adolescent confidentiality and can act as a barrier in seeking and receiving appropriate medical services. Evidence highlights that adolescents who are assured of confidentiality have a greater willingness to discuss sensitive information like substance use, mental health, and sexual history[34,35]. Recently, MOHFW released guidelines to set up a model AFHC in each district hospital, where all the doors and windows of the clinic must have curtains[36]. This low-cost

For peer review only - http://bmjopen.bmj.com/site/about/guidelines.xhtml

approach can be scaled up in all AFHCs irrespective of the level of the health system where the areas for clinical, counseling, and commodity disbursement can be separated by curtains.

These AFHCs are seen as crucial ‘information hubs’ that cater to a diverse range of adolescent health needs. IEC is a powerful tool to bring about social behavior change and promote a culture of ‘health-seeking behavior’ among adolescents, with an emphasis on health promotion and prevention[37]. Therefore, the absence of IEC materials, specifically in the regional language at the health facility, can act as a hurdle in utilization of the health services by adolescents. Studies across various Indian states including Gujarat (Ahmedabad)[25], Rajasthan[29] replicate the finding of limited IEC material at the AFHCs, while AFHCs in West Bengal[28] provided IEC material on Sexual and Reproductive Health (SRH). Thus, the key is to have a combination of no-tech and high-tech resources in AFHCs that may assist in catering to a wider range of literacy levels of adolescents. No tech can include printed IEC materials such as posters, flipbooks[37], and activity books and high tech can include animated movies, short bites on adolescent health issues in the clinic waiting area as seen in clinics of Sweden[38] and Ghana[39].

Additional lacunae include the lack of guidelines on services, such as referral guidelines further limiting the utilization of quality services. These findings are consistent with other national [30] and international studies[40,41] that lack guidelines and SOPs in most AFHCs. These guidelines are a roadmap to a strong connection between various healthcare systems, ensuring efficient use of medical services. The introduction of referral guidelines enhances effective service delivery by reducing patient waiting time, overcrowding and improving patient in-and-out flow. It is also anticipated to raise the bar for patient management by more appropriate use of investigations, better use of patient counseling, and other patient-specific program services[42].

WHO global standard of quality health services for adolescents

For peer review only - http://bmjopen.bmj.com/site/about/guidelines.xhtml

Besides, the aforementioned strengths and limitations of AFHCs, identified using national benchmarks, the study also assessed the compliance of AFHCs to WHO global standards for adolescent-friendly health services. In addition, to some of the overlapping observations with the RKSK benchmarks, findings related to the WHO standards suggest a needed improvement in the observed AFHCs on clear and defined job descriptions, guidelines and SOPs. The majority of the AFHCs included in the study lacked clearly defined job descriptions for medical officers, counselors, nurse/ANM etc and thus might contribute to ambiguity in the delivery of services. To support this finding, a recent assessment in Bhutan highlighted that more than half of healthcare providers (58%) did not provide SRH-related services to adolescents and 74% said their facilities did not have a guideline or an SOP to provide equal and standard services to adolescents[2]. Similarly, a study in Ahmedabad (India) reveals shortcomings in the knowledge and technical capabilities of healthcare providers in areas related to health promotion, disease prevention, and management for adolescents[25].

Similarly, in AFHCs observed in two study states, the rights of adolescents to information, including privacy, confidentiality, non-discrimination, and non-judgemental attitude were not on display, hindering the structural quality. Likewise, studies from India[29,32] and across the globe have reported judgemental and discriminatory behaviour[43,44], and breach of confidentiality[43] by healthcare providers. Strong policies and protocols can enhance the quality of healthcare in India as evident in Ghana[39] where adolescents' positive perception of quality healthcare was influenced by fair, non-discriminatory, respectful, competent and trustworthy healthcare providers. Compliance at the global level, therefore, emphasizes the need for organizing training programs such as those conducted in Tanzania[45] to improve the youth friendliness of the AFHCs and to ensure that these practices are consistently upheld protocols, guidelines, and SOPs in AFHCs. Displaying written statements can help alleviate fear and confusion about services and adolescent rights.[46]

For peer review only - http://bmjopen.bmj.com/site/about/guidelines.xhtml

Moreover, while most of the recruited AFHCs had clean surroundings and proper signage; the absence of other infrastructural characteristics in the two AFHCs when compared to WHO standards, such as clean drinking water, safe storage, appropriate disposal of waste, functioning equipment and medical supplies need maintenance for effective healthcare delivery. The need for infrastructural and medical supplies for effective and appropriate service delivery and adolescent satisfaction has been substantiated in the assessment of services across countries [39,47]. The finding of insufficient medical supplies and equipment is congruent with facilities in Rajasthan (India)[29] and Uganda[41], Nigeria[48] while contradicting the AFHC in Dehana district, Ethiopia[40] where availability of facility characteristics was found to be appropriate. Further, to support quality improvement adolescent participation holds prime importance, as healthcare services should be participant-sensitive and reflect their needs.

The study findings also showed that the unavailability of guidelines on informed consent to assess adolescent participation, is similar to facilities in the East and Southern Africa Region including[39] and those in Comoros, Mozambique, Zambia, etc., suggesting advocating for the development of youth-responsive health systems rather than just providing health care that is friendly to young people[49].

Going forward, fulfilling certain aspects of national benchmarks is indicative of the Government of India's commitment to strengthening adolescent health. Nevertheless, there is a need to strengthen the existing AFHC as the lack of access to youth-friendly services paves the way for risky health behaviors among adolescents and exposes them to numerous vulnerabilities[50]. The AFHCs can be strengthened using the approach of community engagement and participatory co-design with youth at the centre to maximize access, uptake and coverage[51,52] by fostering ownership and assessing the impact of this approach in India.

For peer review only - http://bmjopen.bmj.com/site/about/guidelines.xhtml

This study provides data on the standard of adolescent-friendly health care in public health facilities in India. The findings should be interpreted with caution due to the limited geographical scope of the study. The sample included one clinic from each level of the health system in the selected study districts and thus may not be generalizable to the entire country. To have a more comprehensive picture, it would have been beneficial to conduct exit interviews with adolescent clients and interviews with community members in future research.

Conclusion

This study provides a window of opportunity for improvement by identifying few gaps in existing functional AFHCs. It is essential to address these gaps to create adolescent-friendly health facilities that can contribute to the survival, well-being and transformative growth of adolescents in India. Taking cohesive actions by involving multiple stakeholders and using an approach of community engagement and participatory co-design is crucial to ensure that health services become acceptable, equitable, and accessible to all adolescents.

Patient and Public Involvement

Patients or the public were not involved in the design, conduct, reporting, or dissemination plans of our research.

Funding statement: This work was supported by Medical Research Council, UK grant number MC_PC_MR/P011446/1.

Competing Interests: None declared

Authors' Contribution: MA, SB and DB conceptualized this study. DB and HM were involved in data collection. DB, SB, HM, SK, AMJ and AM were involved in the analysis and interpretation of the findings. DB, SB, AM and HM drafted the manuscript. MA, AMJ and SD, critically reviewed the manuscript.

For peer review only

A data sharing statement: Data are available on reasonable request. As per our Institutional's data sharing policy, prior approval is required from the Research Management Committee (RMC) and the Principal Investigator (PI) of the study. After approval of request from the Committee, de-identified data can only be shared upon request.

Acknowledgments: We would like to express our sincere gratitude to all the staff of the Adolescent Friendly Health Clinics in Maharashtra and Madhya Pradesh included in this study. We also thank Ms. Gayatri Nayak, Consultant (Public Health Foundation of India) to facilitate the data collection.

For peer review only

References

1. World Health Organisation. Making health services adolescent friendly: developing national quality standards for adolescent friendly health services. 2012. Available: https://apps.who.int/iris/bitstream/handle/10665/75217/9789241503594_eng.pdf
2. World Health Organisation. Rapid assessment of the implementation of adolescent health programmes in countries of South-East Asia - Regional Summary. 2021. Available: https://apps.who.int/iris/bitstream/handle/10665/342587/9789290228639-eng.pdf?sequence=1&isAllowed=y
3. Ninsiima LR, Chiumia IK, Ndejjo R. Factors influencing access to and utilisation of youth-friendly sexual and reproductive health services in sub-Saharan Africa: a systematic review. *Reprod Health* 2021;**18**(135). doi:10.1186/s12978-021-01183-y
4. Ministry of Health and Family Welfare, Government of India. Adolescent Health (RKSK): National Health Mission. Available: https://www.nhm.gov.in/index1.php?lang=1&level=2&sublinkid=818&lid=221 [Accessed 24 Dec 2023]
5. Ministry of Health and Family Welfare, Government of India. Implementation

For peer review only - http://bmjopen.bmj.com/site/about/guidelines.xhtml

Guidelines Rashtriya Kishor Sawasthya Karyakram(RKSK). 2018. Available: https://nhm.gov.in/New_Updates_2018/NHM_Components/RMNCHA/AH/guidelines/Implementation_Guidelines_Rashtriya_Kishor_Swasthya_Karyakram(RKSK)_2018.pdf

6. Adolescent Health Division. Ministry of Health and Family Welfare, Government of India. Operational framework- Translating strategy into programmes. 2014. Available: https://nhm.gov.in/images/pdf/programmes/RKSK/RKSK_Operational_Framework.pdf

7. Santhya K, Acharya R, Pandey N, *et al*. Understanding the Lives of Adolescents and Young Adults (UDAYA) in Bihar, India 2017. Available: https://www.projectudaya.in/wp-content/uploads/2018/08/UP-Report-pdf.pdf

8. Sowmya H, Singhe MS. Utilization of Adolescent Friendly Health Clinic (AFHC) services- A study of Dakshina Kannad district . *JAC: A J Compos Theory* 2020;**13**(10).

9. Mahalakshmy T, Premarajan KC, Soundappan K, *et al*. A mixed methods evaluation of adolescent friendly health clinic under national adolescent health program, Puducherry, India. *Indian J Pediatr* 2019;**86**(2):132-139. doi:10.1007/S12098-018-2755-4

10. Singh A, Joshi HS, Upadhyay D, *et al*. A study on awareness regarding treatment offered in adolescent-friendly health services in the rural area of Bareilly district. *Int J Adv Integr Med Sci* 2018;**3**(1):12-15. doi:10.5005/JP-JOURNALS-10050-10113

11. Wadhwa R, Chaudhary N, Bisht N, *et al*. Improving adolescent health services across high priority districts in 6 states of India: Learnings from an Integrated Reproductive Maternal Newborn Child and Adolescent Health Project. *Indian J Community Med* 2018;**43**(Suppl 1):S6. doi:10.4103/IJCM.IJCM_38_18

12. Chingono RMS, Mackworth-Young CRS, Ross DA, *et al*. Designing routine health

For peer review only

checkups for adolescents in Zimbabwe. *J Adolesc Health* 2021;**69**(6):940-947. doi: 10.1016/j.jadohealth.2021.07.002

13. World Health Organization, UNAIDS. Global standards for quality health-care services for adolescents: a guide to implement a standards-driven approach to improve the quality of health care services for adolescents. Volume 1: Standards and criteria. World Health Organization. 2015. Available: https://apps.who.int/iris/bitstream/handle/10665/183935/9789241549332_vol1_eng.pdf?sequence=1&isAllowed=y

14. Ministry of Health & Family Welfare, Government of India. Adolescent Friendly Health Clinics (AFHCs): National Health Mission. Available: https://nhm.gov.in/index1.php?lang=1&level=3&sublinkid=1247&lid=421 [Accessed 19 July 2021].

15. Centers for Disease Control and Prevention. US Department of Health and Human Services. Data Collection Methods for Program Evaluation: Observation. 2018. Available: https://www.cdc.gov/healthyyouth/evaluation/pdf/brief16.pdf

16. World Health Organization. Adolescent health. Available: https://www.who.int/health-topics/adolescent-health#tab=tab_1 [Accessed 2 Aug 2022]

17. Government of India Ministry of Home Affairs. Census of India | Office of the Registrar General & Census Commissioner, India. 2011. Available: https://censusindia.gov.in/census.website/ [Accessed 23 Aug 2022].

18. Arora M, Dringus S, Bahl D, *et al*. Engagement of health workers and peer educators from the National Adolescent Health Programme-Rashtriya Kishor Swasthya Karyakram during the COVID-19 pandemic: Findings from a situational analysis. *PLoS One* 2022;**17**(9 September):1-18. doi:10.1371/journal.pone.0266758

19. Steps in Applying Probability Proportional to Size (PPS) and Calculating Basic

For peer review only - http://bmjopen.bmj.com/site/about/guidelines.xhtml

Probability Weights. Available: https://cdn.who.int/media/docs/default-source/hq-tuberculosis/global-task-force-on-tb-impact-measurement/meetings/2008-03/p20_probability_proportional_to_size.pdf?sfvrsn=51372782_3

20. Chuenjitwongsa S. How to: conduct a delphi study. 2017. Available:https://www.cardiff.ac.uk/__data/assets/pdf_file/0010/1164961/how_to_conduct_a_delphistudy.pdf

21. World Health Organization, UNAIDS. Global standards for quality health-care services for adolescents: a guide to implement a standards-driven approach to improve the quality of health care services for adolescents. Volume 3: Tools to conduct quality and coverage measurement surveys to collect data about compliance with the global standards. World Health Organization. 2015. Available: https://apps.who.int/iris/bitstream/handle/10665/183935/9789241549332_vol3_eng.pdf?sequence=5&isAllowed=y

22. Centers for Disease Control and Prevention. Epi Info. Available: https://www.cdc.gov/epiinfo/index.html [Accessed 2 Aug 2022]

23. World Health Organization, UNAIDS. Global standards for quality health-care services for adolescents: a guide to implement a standards-driven approach to improve the quality of health care services for adolescents. Volume 4: Scoring sheets for data analysis. World Health Organization. 2015. Available: https://apps.who.int/iris/bitstream/handle/10665/183935/9789241549332_vol4_eng.pdf?sequence=6&isAllowed=y

24. Dolma Y. Rapid assessment of adolescent health clinic at sub-district hospital of Leh. *GJRA - Glob J Res Anal* 2018;**7**(2):141-142.

25. Dixit GT, Jain S, Mansuri F, *et al*. Adolescent friendly health services : where are we actually standing ? *Int J Community Med Public Heal* 2017;**4**(3):820-824.

For peer review only - http://bmjopen.bmj.com/site/about/guidelines.xhtml

26. Bhat AA, Jan Y, Jan R, *et al*. Adolescent friendly health centres: A review from North India. *IOSR Journal of Dental and Medical Sciences (IOSR-JDMS)* 2019;**18**(8):44-48. doi:10.9790/0853-1808044448

27. Bali S, Yadav K, Alok Y. A study of physical infrastructure and preparedness of Public Health Institution for providing adolescent friendly health services in Central India. *Indian J Prev Soc Med* 2020;**51**(4):195-203.

28. Biswas T, Majumdar KK. A study of the functioning of the adolescent friendly health clinics as well as the awareness and health seeking behaviour of adolescents attending the adolescent friendly health clinics in the Rampurhat health district, West Bengal, India. *Int J Community Med Public Health* 2022;**9**(11):4124-4131. doi:10.18203/2394-6040.ijcmph20222907

29. Dayal R, Gundi M. Assessment of the quality of sexual and reproductive health services delivered to adolescents at Ujala clinics: A qualitative study in Rajasthan, India. *PLoS One* 2022;**17**(1). doi:10.1371/JOURNAL.PONE.0261757

30. Awang H, Rahman AA, Sukeri S, *et al*. Making health services adolescent-friendly in Northeastern Peninsular Malaysia : A mixed-methods study. *Int J Environ Res Public Heal* 2020;**17**(4):1341. doi:10.3390/ijerph17041341

31. Daley AM, Polifroni EC, Sadler LS. The essential elements of adolescent-friendly care in school-based health centers : A mixed methods study of the perspectives of nurse practitioners and adolescents. *J Pediatr Nurs* 2019;**47**:7-17. doi:10.1016/j.pedn.2019.03.005

32. Santhya KKG, Prakash R, Jejeebhoy SJ, et al. Accessing adolescent friendly health clinics in India: The perspectives of adolescents and youth. 2014. Available: https://www.popcouncil.org/uploads/pdfs/2014PGY_AFHC-IndiaReport.pdf

33. Yadav RJ, Mehta R, Pandey A, *et al*. Evaluation of adolescent-friendly health services

For peer review only - http://bmjopen.bmj.com/site/about/guidelines.xhtml

in India. *Heal Popul Perspect Issues* 2009;**32**(2):96-104.

34. Ford CA, Thomsen SL, Compton B. Adolescents' interpretations of conditional confidentiality assurances. *J Adolesc Health*. 2001;29(3):156-159. doi:10.1016/s1054-139x(01)00251-8.

35. Ford CA, Millstein SG, Halpern-Felsher BL, *et al*. Influence of physician confidentiality assurances on adolescents' willingness to disclose information and seek future health care. A randomized controlled trial. *JAMA.* 1997;278:1029–34. doi: 10.1001/jama.1997.03550120089044.

36. Ministry of Health and Family Welfare, Government of India. Guidelines for Operationalizing Model Adolescent Friendly Health Clinics (M-AFHC). 2022. Available: https://nhm.gov.in/images/pdf/programmes/RKSK/Guidelines_(M-%20AFHRCs).pdf

37. Sharma S, Akhtar F, Singh RK, *et al*. Validation of health education material for youth: A step to ensure implementation fidelity in community-based interventions. *Healthcare (Basel)* 2019;**8(1)**:8. doi:10.3390/healthcare8010008.

38. Thomée S, Malm D, Christianson M, *et al*. Challenges and strategies for sustaining youth-friendly health services — a qualitative study from the perspective of professionals at youth clinics in northern Sweden. *Reprod Health* 2016;**13**(1):147. doi:10.1186/s12978-016-0261-6

39. Anaba, EA, Abuosi, AA. Assessing health care quality in adolescent clinics , implications for quality improvement. *Int J Heal Gov* 2019;(August 2018). doi:10.1108/IJHG-03-2018-0012

40. Gebrie M, Asrade G, Tsehay CT, *et al*. Quality of adolescent and youth-friendly health services in Dehana district public health facilities , northeast Ethiopia : Using the Donabedian quality framework. *PLoS One* 2021;**16**(10).

For peer review only - http://bmjopen.bmj.com/site/about/guidelines.xhtml

doi:10.1371/journal.pone.0258718

41. Bukenya JN, Mulogo E, Kibira SPS, *et al*. Health facilities' readiness to provide friendly reproductive health services to young people aged 10-24 years in Wakiso district, Uganda. *Glob J Reprod Med* 2018;**2**(3). doi:10.19080/GJORM.2017.02.555588.Health

42. Department of Medical, Health and Family Welfare, Lucknow. Referral guidelines for health facilities in Uttar Pradesh. Available: http://dgmhup.gov.in/Documents/Referral_Guidelines.pdf

43. Geary RS, Gómez-olivé FX, Kahn K, *et al*. Barriers to and facilitators of the provision of a youth-friendly health services programme in rural South Africa. *BMC Health Serv Res* 2014:1-8. doi: 10.1186/1472-6963-14-259

44. Chilinda I, Hourahane G, Pindani M, *et al*. Attitude of health care providers towards adolescent sexual and reproductive health services in developing countries : A Systematic Review. *Health (Irvine Calif)* 2014;(July):1706-1713. doi: 10.4236/health.2014.614203

45. Renju J, Andrew B, Nyalali K, *et al*. A process evaluation of the scale up of a youth-friendly health services initiative in northern Tanzania. *J Int AIDS Soc* 2010;**13**:32. doi:10.1186/1758-2652-13-32

46. Importance of SOPs Urban Primary Health and Wellness Centre**.** Available: https://vikaspedia.in/health/health-care-innovations/good--replicable-and-innovative-practices/importance-of-sops-urban-primary-health-and-wellness-centre. [Accessed 26 March 2023].

47. United Nations Population Fund, International Planned Parenthood Federation. Regional report: Assessment of adolescents and youth-friendly health service delivery in the East and Southern Africa region.2017. Available:

For peer review only

For peer review only - http://bmjopen.bmj.com/site/about/guidelines.xhtml

https://esaro.unfpa.org/sites/default/files/pub-pdf/Assessment of Adolescents and Youth-Friendly Health Service Delivery in ESA.pdf

48. Osanyin Y. Report on assessment of facilities providing youth friendly health services in Nigeria. 2013. Available: http://www.ncceonline.edu.ng/download/files/Assessment of Youth Friendly Health Centers and Services.pdf

49. Sheikh K, Ranson MK, Gilson L. Explorations on people centredness in health systems. *Health Policy Plan* 2014;**29**:II1-II5. doi:10.1093/heapol/czu082

50. Partnership for Maternal, Newborn & Child Health. Ministry of Health and Family Welfare, Government of India. Centre staging health and well-being of adolescents and young people. 2023. Available: https://pmnch.who.int/docs/librariesprovider9/governance/g20-event-technical-session-1.pdf?sfvrsn=8f8ff09b_5

51. Ministry of Health and Family Welfare, Government of India. Available: https://pib.gov.in/PressReleasePage.aspx?PRID=1933647 [Accessed 6 Aug 2023]

52. Mackworth-Young C, Dringus S, Dauya E, *et al.* Putting youth at the centre: co-design of a community-based intervention to improve HIV outcomes among youth in Zimbabwe [version 2; peer review: 1 approved]. *Wellcome Open Res* 2022;**7**:53. https://doi.org/10.12688/wellcomeopenres.17531.2

For peer review only

For peer review only - http://bmjopen.bmj.com/site/about/guidelines.xhtml

Table S1: Compliance of Adolescent Friendly Health Clinics (AFHCs) with RKSK Benchmarks: Item-Wise Scores.

**Items Under Each Benchmark**	**Method of asessment**<br>**Observation /**<br>**Observation and verified by interview**	**AFHCs with Presence of item**<br>**N (%)**
**Infrastructure- clean, bright and colorful (Max. Marks= 5)**		
Overall infrastructure of AFHC is bright with wall paints, color, furniture	Observation	6 (42.9)
Is the toilet clean?	Observation	5 (35.7)
Are the surroundings of the facility clean?	Observation	11 (78.5)
Does the waiting area have adequate lighting?	Observation	9 (64.3)
Does the waiting area seem clean overall?	Observation	8 (57.1)
**Can be easily accessed by the adolescents (distance and convenient working hours) (Max. Marks= 3)**		
Do the functional days of the AFHC match with the RKSK operational framework?	Observation and verified by Interview	11 (78.5)
Do the Operational Hours of the AFHC match with the RKSK operational framework?	Observation and verified by Interview	11 (78.5)
Is AFHC accessible to adolescents i.e. where adolescents can reach using the local transport?	Observation	14 (100)
**Awareness about the Clinic and its range of services (IEC, proper signage etc.) (Max. Marks= 6)**		
Signboard mentions that it is the AFHC	Observation	10 (71.4)
Signboard that mentions the facility's operating hours?	Observation	8 (57.1)
Signboard clearly visible?	Observation	8 (57.1)
Signboard displayed in the regional language?	Observation	7 (50)
Is the IEC material displayed in regional language?	Observation	5 (35.7)
Does the waiting area have information, education and communication materials specifically developed for adolescents?	Observation	6 (42.9)

For peer review only

**Maintains privacy and confidentiality (Max. Marks= 10)**		
Is the waiting area in a common area ?	Observation	8 (57.1)
AFHC is separated from general OPD	Observation	10 (71.4)
Separate area for Counselling services	Observation	9 (64.3)
Separate area for Clinical services	Observation	4 (28.6)
Separate area for commodities disbursement	Observation	5 (35.7)
There are curtains on the doors and windows	Observation	8 (57.1)
Case records are kept in a secure place, accessible only to authorized personnel	Observation and verified by Interview	13 (92.9)
The registers are kept under lock and key outside operating hours	Observation and verified by Interview	11 (78.5)
Information on the identity of the adolescent and the presenting issue are gathered in confidence during registration	Observation and verified by Interview	9 (64.3)
No one can see or hear an adolescent client from the outside during the consultation or counselling	Observation	9 (64.3)
**Referral from the periphery/community and further referral linkages with the higher facilities and specialty clinics. (Max. Marks= 1)**		
Referral guidelines	Observation and verified by interview	1 (7.1)

For peer review only

Table S2: Compliance of Adolescent Friendly Health Clinics (AFHCs) with WHO Global standards for quality health-care services for adolescents: Item-Wise Scores.

**Items Under Each Standard**	**Method of assessment**<br>**Observation**<br>**Observation and verified by interview**	**Yes**
**Standard 1- Adolescents' health literacy (Max. Score= 3)**		
Signboard that mentions the facility's operating hours?	Observation	8 (57.1)
Signboard clearly visible?	Observation	8 (57.1)
Does the waiting area have information, education and communication materials specifically developed for adolescents?	Observation	6 (42.9)
**Standard 3- Appropriate package of services (Max. Score= 2)**		
Guidelines/SOPs for which services should be provided in the facility and in the community	Observation and verified by interview	0 (0)
Referral guidelines	Observation and verified by interview	1 (7.1)
**Standard 4- Providers' competencies (Max. Score= 10)**		
BMI growth charts for adolescents	Observation	3 (21.5)
The rights of adolescents to information, non-judgmental attitude and respectful care	Observation and verified by interview	1 (7.1)
The policy commitment of the health facility to provide health services to all adolescents without discrimination	Observation and verified by interview	1 (7.1)
The policy on confidentiality and privacy	Observation and verified by interview	0 (0)
The policy and procedure to ensure free or affordable service provision for adolescents	Observation and verified by interview	0 (0)
Job Description- Medical Officer	Observation and verified by interview	1 (7.1)

For peer review only

Job Description- Staff Nurse	Observation and verified by interview	0 (0)
Job Description- ANM	Observation and verified by interview	0 (0)
Job Description- Counsellor	Observation and verified by interview	1 (7.1)
Job Description- Specialist	Observation and verified by interview	0 (0)
**Standard 5- Facility characteristics (Max. Score= 34)**		
Does the waiting area have information, education and communication materials specifically developed for adolescents?	Observation	6 (42.9)
Does the waiting area have drinking water?	Observation	0 (0)
Does the waiting area seem welcoming overall?	Observation	6 (42.9)
Does the waiting area seem clean overall?	Observation	8 (57.1)
Is the toilet clean?	Observation	5 (35.7)
Does the facility have permanent electricity during working hours?	Observation	9 (64.3)
Does the facility have safe waste disposal?	Observation	3 (21.5)
Does the facility have safe storage and disposal of sharps?	Observation	3 (21.5)
Does the facility have adequate hand hygiene facilities that are located in or adjacent to the office/examination room?	Observation	6 (42.9)
Does the toilet have functioning hand hygiene facilities?	Observation	6 (42.9)
Are the surroundings of the facility clean?	Observation	11 (78.5)
Blood pressure measurement machine	Observation	2 (14.3)
Stethoscope	Observation	3 (21.5)

For peer review only

Clinical thermometer	Observation	2 (14.3)
Weighing scale	Observation	10 (71.4)
Measuring tape	Observation	1 (7.1)
Height Meter/Stadiometer	Observation	2 (14.3)
Hemoglobin Testing	Observation	3 (21.5)
Provision of Condoms	Observation and verified by interview	6 (42.9)
Provision of Oral Contraceptive Pills	Observation and verified by interview	3 (21.5)
Provision of Emergency Contraceptive Pills	Observation and verified by interview	1 (7.1)
Provision of Pregnancy Testing Kits	Observation and verified by interview	2 (14.3)
Paracetamol	Observation and verified by interview	3 (21.5)
Guidelines/SOPs on protecting the privacy and confidentiality of adolescents	Observation and verified by interview	0 (0)
Guidelines/SOPs on including staff responsibilities for making the health facility welcoming, convenient and clean	Observation and verified by interview	0 (0)
SOPs on how to minimize waiting time	Observation and verified by interview	0 (0)
SOPs on how to provide services to adolescents with or without an appointment	Observation and verified by interview	0 (0)
There are curtains on the doors and windows	Observation	8 (57.1)
Communication between reception staff and visitors is private and cannot be overheard, including from the waiting room	Observation	2 (14.3)
Case records are kept in a secure place, accessible only to authorized personnel	Observation and verified by interview	13 (92.9)
The registers are kept under lock and key outside operating hours	Observation and verified by interview	11 (78.5)

For peer review only

Information on the identity of the adolescent and the presenting issue are gathered in confidence during registration	Observation	9 (64.3)
In the offices/examining rooms, there is a screen to separate the examination area from the consultation area	Observation	4 (28.6)
No one can see or hear an adolescent client from the outside during the consultation or counselling	Observation	9 (64.3)
**Standard 6- Equity and non-discrimination (Max. Score= 4)**		
The policy commitment of the health facility to provide health services to all adolescents without discrimination	Observation and verified by interview	1 (7.1)
The policy and procedure to ensure free or affordable service provision for adolescents	Observation and verified by interview	0 (0)
Guidelines/SOPs on applying policies for free, or affordable, service provision to adolescents	Observation and verified by interview	0 (0)
Guidelines/SOPs on equitable service provision to all adolescents irrespective of their ability to pay, age, sex, marital status or other characteristics	Observation and verified by interview	0 (0)
**Standard 7- Data and quality improvement (Max. Score= 3)**		
Enrolment Register	Observation and verified by interview	14 (100)
Counselling Register	Observation and verified by interview	12 (85.7)
Stock Register	Observation and verified by interview	2 (14.3)
**Standard 8- Adolescents' participation (Max. Score= 1)**		
Guidelines/SOPs on informed consent	Observation and verified by interview	0 (0)

For peer review only

SRQR Reporting checklist for qualitative study.

Instructions to authors

Complete this checklist by entering the page numbers from your manuscript where readers will find each of the items listed below. SRQR reporting guidelines: O'Brien BC, Harris IB, Beckman TJ, Reed DA, Cook DA. Standards for reporting qualitative research: a synthesis of recommendations. Acad Med. 2014;89(9):1245-1251.

		Reporting Item	Page Number
**Title**			
	#1	Concise description of the nature and topic of the study identifying the study as qualitative or indicating the approach (e.g. ethnography, grounded theory) or data collection methods (e.g. interview, focus group) is recommended	Page 1
**Abstract**			Abstract
	#2	Summary of the key elements of the study using the abstract format of the intended publication; typically includes background, purpose, methods, results and conclusions	Page 1
**Introduction**			
Problem formulation	#3	Description and signifcance of the problem / phenomenon studied: review of relevant theory and empirical work; problem statement	Introduction Page 3-5
Purpose or research question	#4	Purpose of the study and specific objectives or questions	Introduction Page 5
**Methods**			
Qualitative approach and research paradigm	#5	Qualitative approach (e.g. ethnography, grounded theory, case study, phenomenolgy, narrative research) and guiding theory if appropriate; identifying the research paradigm (e.g. postpositivist, constructivist / interpretivist) is also recommended; rationale. The rationale should briefly discuss the justification for choosing that theory, approach, method or technique rather than other options available; the assumptions and limitations implicit in those choices and how those choices influence study conclusions and transferability. As appropriate the rationale for several items might be discussed together.	Data collection, Page 7<br>Data analysis: Page 8, Para 1
Researcher characteristics and reflexivity	#6	Researchers' characteristics that may influence the research, including personal attributes, qualifications / experience, relationship with participants, assumptions and / or presuppositions; potential or actual interaction between researchers' characteristics and the research questions, approach, methods, results and / or transferability	Data collection, page 7
Context	#7	Setting / site and salient contextual factors; rationale	Study setting and Sample selection Page 5
Sampling strategy	#8	How and why research participants, documents, or events were selected; criteria for deciding when no further sampling was necessary (e.g. sampling saturation); rationale	Study setting and sample selection Page 7
Ethical issues pertaining to human subjects	#9	Documentation of approval by an appropriate ethics review board and participant consent, or explanation for lack thereof; other confidentiality and data security issues	Page 8, Para 2 and Para 3
Data collection methods	#10	Types of data collected; details of data collection procedures including (as appropriate) start and stop dates of data collection and analysis, iterative process, triangulation of sources / methods, and modification of procedures in response to evolving study findings; rationale	Data Collection Page 7, 8

For peer review only - http://bmjopen.bmj.com/site/about/guidelines.xhtml

Data collection instruments and technologies	#11	Description of instruments (e.g. interview guides, questionnaires) and devices (e.g. audio recorders) used for data collection; if / how the instruments(s) changed over the course of the study	Data collection and procedure: Page 7
Units of study	#12	Number and relevant characteristics of participants, documents, or events included in the study; level of participation (could be reported in results)	Results: Page 13, 14
Data processing	#13	Methods for processing data prior to and during analysis, including transcription, data entry, data management and security, verification of data integrity, data coding, and anonymisation / deidentification of excerpts	Measures, Page 8, Data analysis: Page 12
Data analysis	#14	Process by which inferences, themes, etc. were identified and developed, including the researchers involved in data analysis; usually references a specific paradigm or approach; rationale	Data analysis: Page 12
Techniques to enhance trustworthiness	#15	Techniques to enhance trustworthiness and credibility of data analysis (e.g. member checking, audit trail, triangulation); rationale	Data analysis: Page 12
**Results/findings**			
Syntheses and interpretation	#16	Main findings (e.g. interpretations, inferences, and themes); might include development of a theory or model, or integration with prior research or theory	Results: Page 14-20
Links to empirical data	#17	Evidence (e.g. quotes, field notes, text excerpts, photographs) to substantiate analytic findings	NA
**Discussion**			
Intergration with prior work, implications, transferability and contribution(s) to the field	#18	Short summary of main findings; explanation of how findings and conclusions connect to, support, elaborate on, or challenge conclusions of earlier scholarship; discussion of scope of application / generalizability; identification of unique contributions(s) to scholarship in a discipline or field	Discussion: Page 21-25
Limitations	#19	Trustworthiness and limitations of findings	Discussion: Page 25
**Other**			
Conflicts of interest	#20	Potential sources of influence of perceived influence on study conduct and conclusions; how these were managed	Page 25
Funding	#21	Sources of funding and other support; role of funders in data collection, interpretation and reporting	Page 25

None The SRQR checklist is distributed with permission of Wolters Kluwer © 2014 by the Association of American Medical Colleges. This checklist can be completed online using https://www.goodreports.org/, a tool made by the EQUATOR Network in collaboration with Penelope.ai

For peer review only

BMJ Open

Compliance of Adolescent-Friendly Health Clinics with National and International Standards: Quantitative Findings from the i-Saathiya Study

Journal:	*BMJ Open*
Manuscript ID	bmjopen-2023-078749.R1
Article Type:	Original research
Date Submitted by the Author:	11-Jan-2024
Complete List of Authors:	Bahl, Deepika; Public Health Foundation of India, Health Promotion Division<br>Bassi, Shalini; Public Health Foundation of India, Health Promotion Division<br>Maity, Heeya; Public Health Foundation of India<br>Krishnan, Supriya ; Public Health Foundation of India<br>Dringus, Stefanie; independent global health consultant<br>Mason-Jones, Amanda; University of York, Health Sciences<br>Malik, Anku; Public Health Foundation of India<br>Arora, Monika; Public Health Foundation of India, Health Promotion Division
**Primary Subject Heading**:	Public health
Secondary Subject Heading:	Public health, Health services research
Keywords:	Adolescent, Health Services Accessibility, PUBLIC HEALTH

SCHOLARONE™
Manuscripts

I, the Submitting Author has the right to grant and does grant on behalf of all authors of the Work (as defined in the below author licence), an exclusive licence and/or a non-exclusive licence for contributions from authors who are: i) UK Crown employees; ii) where BMJ has agreed a CC-BY licence shall apply, and/or iii) in accordance with the terms applicable for US Federal Government officers or employees acting as part of their official duties; on a worldwide, perpetual, irrevocable, royalty-free basis to BMJ Publishing Group Ltd ("BMJ") its licensees and where the relevant Journal is co-owned by BMJ to the co-owners of the Journal, to publish the Work in this journal and any other BMJ products and to exploit all rights, as set out in our licence.

The Submitting Author accepts and understands that any supply made under these terms is made by BMJ to the Submitting Author unless you are acting as an employee on behalf of your employer or a postgraduate student of an affiliated institution which is paying any applicable article publishing charge ("APC") for Open Access articles. Where the Submitting Author wishes to make the Work available on an Open Access basis (and intends to pay the relevant APC), the terms of reuse of such Open Access shall be governed by a Creative Commons licence – details of these licences and which Creative Commons licence will apply to this Work are set out in our licence referred to above.

Other than as permitted in any relevant BMJ Author's Self Archiving Policies, I confirm this Work has not been accepted for publication elsewhere, is not being considered for publication elsewhere and does not duplicate material already published. I confirm all authors consent to publication of this Work and authorise the granting of this licence.

For peer review only

Title: Compliance of Adolescent-Friendly Health Clinics with National and International Standards: Quantitative Findings from the i-Saathiya Study

Authors: Deepika Bahl[1], Shalini Bassi[1], Heeya Maity[1], Supriya Krishnan[1], Stefanie Dringus[2], Amanda J. Mason-Jones[3], Anku Malik[1], Monika Arora[1]

Affiliations

[1]Health Promotion Division, Public Health Foundation of India, Delhi, India.

[2]Independent Global Health Consultant, London, England.

[3]Department of Health Sciences, University of New York, Heslington, York, England.

Corresponding Author*

Dr. Deepika Bahl

Email: deepika.bahl@phfi.org

For peer review only

Abstract

Objective: Indian adolescents experience several health challenges requiring acceptable, equitable, appropriate, and effective healthcare services. Our objective was to assess the compliance of Adolescent Friendly Health Clinics (AFHCs) in two of India's largest states, using both national benchmarks (under Rashtriya Kishor Swasthya Karykaram-RKSK) and global standard (by World Health Organization).

Design: Cross-sectional study comprising structured observations and interactions was conducted (November 2021 to June 2022).

Setting: Fourteen AFHCs across all levels of health system were included from two districts of Maharashtra (n=8) and Madhya Pradesh (n=6). These AFHCs were observed using checklist, and few items of checklist were verified by interactions with AFHC's health workers (Medical Officers/Auxillary Nurse Midwives/ Counsellors) handlings adolescents. The developed checklist included 57 items based on adapted global standards and national benchmarks.

Result: High compliance of AFHCs with RKSK's benchmarks was attributed to various items including the accessibility through local transport (n=14, 100%), clean surroundings (n=11, 78.5%), presence of signage (n=10, 71.4%), convenient operating days and time (n=11, 78.5%), and secure storage of records (n=13, 92.9%). Concurrently, items that showed low compliance encompassed, the availability of Information, Education and Communication resources, which were deficient in 57.1% of AFHCs (n=8). Similarly, designated areas for

For peer review only - http://bmjopen.bmj.com/site/about/guidelines.xhtml

clinical services (n=10, 71.4%) and commodity disbursement (n=9, 64.3%) lacked in more than half of the recruited AFHCs. Additionally, lack of guidelines for referrals (n=13, 92.9%), as well as Standard Operating Procedures to ensure equity, non-judgemental attitude, competence, confidentiality and referral as per World Health Organization standards.

Conclusion: Evidence spotlights the strengths and gaps in AFHCs, aligning with, government's priorities on adolescent health. Addressing the identified gaps is crucial to creating healthcare facilities that are adolescent-friendly, easily accessible, and effectively navigate adolescent health challenges. This concerted effort would contribute to their development and transformation, playing a pivotal role in India's progress.

Strengths and limitations of the study

- The study comprehensively assesses the existing quality of adolescent-friendly healthcare services in India in compliance to both, national and global guidelines.
- Since data was gathered only from two Indian states, the generalisability of the findings may be limited.
- Integrating exit interviews with adolescent clients or employing mystery client methodology would have provided a comprehensive understanding of the healthcare worker's performance.

Introduction

Adolescent-Friendly Health Services (AFHS) are evidence-based interventions to address health concerns by offering accessible, acceptable, equitable, and appropriate healthcare services for adolescents.[1] In the South-East Asian Region, countries are prioritizing adolescent health and well-being by scaling AFHS through their national public health systems. Multiple delivery settings and approaches including clinics, school health programs,

For peer review only - http://bmjopen.bmj.com/site/about/guidelines.xhtml

For peer review only

community outreach, partnerships with civil society, development partners and professional bodies are utilised to provide these services.[2] Evidence highlights that these adolescent-friendly services are cost-effective in delivering sexual and reproductive health (SHR) services to adolescents.[3]

In India, AFHS is provided through Adolescent Friendly Health Clinics (AFHCs) as part of 'Rashtriya Kishor Swasthya Karyakram-RKSK' (India's Adolescent Health Strategy) launched in 2014.[4] AFHC is one of the critical pillars of the RKSK that seeks to enable all adolescents to realize their full potential by making informed decisions concerning their health, and by accessing the services and support to implement their decisions. These clinics are meant to provide a range of services listed as clinical and counselling services related to nutrition, injuries, non-communicable diseases, mental health, substance misuse and sexual and reproductive health to adolescents.[5] Integration of such services in the AFHCs are vital for accelerating the progress of adolescent health in India. The AFHCs have been integrated into medical colleges, district and sub-district hospitals, community health centres (CHCs)/ rural hospitals, and primary health centres (PHCs)[6] to provide comprehensive services for adolescents. Despite years of establishment, the available evidence from India highlights the low footfall of adolescents in AFHCs.[7-9] This is partly explained by multiple barriers to utilizing these services including a lack of adolescents' knowledge about the AFHCs in their vicinity,[10] inadequate staffing, untrained service providers, social taboo, inadequate equipment, low priority accorded by Program Managers, and adolescents' perceive that services are restricted to Reproductive and Sexual Health.[11] This results in a missed opportunity in the identification and treatment of health problems, hindering the assisting provided to adolescents in developing positive healthcare-seeking behaviors.[12] This undermines the goal of RKSK to promote adolescents' health and well-being and emphasizes the pressing need for a standardized approach to enhance the quality of care across India,[11]

For peer review only - http://bmjopen.bmj.com/site/about/guidelines.xhtml

ensuring effective utilization by adolescents. With this, as part of the i-Saathiya study, which aims to explore the implementation of the peer education program, we also assessed AFHC compliance with both national [13] and global standards for quality healthcare services catering to adolescents.[14] This assessment was carried out in the states of Madhya Pradesh and Maharashtra (India) through structured observations and interactions-based verifications. This method was suitable to achieve this objective as it allowed us to see all activities directly, rather than solely relying on subjective opinions and perceptions.[15] We envisage that the findings of this study will be useful in strengthening the Indian AFHCs, enabling them to safeguard, promote and improve adolescent health by providing acceptable, equitable, appropriate, and effective healthcare services; and establishing safe and supportive environments to facilitate their growth and development.[16]

Methodology

Study Setting and Sample Selection

AFHCs in two districts of Madhya Pradesh (Panna and Damoh) and Maharashtra (Yavatmal and Nashik) were observed. Panna and Damoh are situated in Madhya Pradesh, a large state in central India with a total population of 72.7 million and a literacy rate of 69.3%.[17] Yavatmal and Nashik are in Maharashtra, which is situated in the Western Peninsular region of India with an approximate population of 112.4 million and an 82.3% literacy rate.[17] The criteria for selection of states and districts have been described in another i-Saathiya publication.[18] Table 1 lists the AFHCs at various levels of the health system in selected study districts and blocks of both states. To assess compliance, we included AFHCs from all the levels in Madhya Pradesh and Maharashtra. At the higher level, the number of AFHCs were limited, thus all were included for the assessment. One AFHC at the district-level (District Hospital -DH) or Government Medical College (GMC), in all four selected study districts (n=4) were included.

For peer review only - http://bmjopen.bmj.com/site/about/guidelines.xhtml

Each district of Maharashtra featured one AFHC at the Sub-district Hospital (SDH, n=2). But at the lower level of health facility, number of AFHC were more than one. Thus, we adopted a random selection approach using a fishbowl method to choose one AFHC each from Community Health Center (CHC) or Rural Hospital (RH) (n=4) and Primary Health Center (PHC) from each study district (n=4). In total, 14 AFHCs were included in our study for assessment using this methodology.

Table 1: Number# of AFHCs functional at various levels of health system in the study districts and blocks

State	District	Blocks	Level of Health Facility				
			Government Medical College	District Hospital	Sub District Hospital*	CHCs/RH under each block	PHCs under each block
Madhya Pradesh	Panna	Panna	No	1	No	1	4
		Ajaygarh				1	4
		Shahnagar				1	2
		Pawai				1	5
	Damoh	Damoh	No	1	No	1	2
		Jabera				1	2
		Patera				1	2
		Patharia				1	2
Maharashtra	Yavatmal	Babulgaon	1	No	No	1	2
		Pusad			1	No	2
		Yavatmal			No	No	2

For peer review only - http://bmjopen.bmj.com/site/about/guidelines.xhtml

For peer review only

		Zari-Jamani			No	1**	2
	Nashik	Dindori	No	1		2** (RH equivalent to CHC)	2
		Nandgaon			1	1**	2
		Sinner				1**	2
		Surgana				2**	2
Total			1	3	2	16	39

#AFHC numbers during November 2021- June 2022

* SDH only exist in Maharashtra

** RH equivalent to CHC

Study design

A cross-sectional study using quantitative methods was conducted between November 2021 and June 2022.

Data collection

The trained study team conducted structured observations using a printed checklist with several performance criteria. This assessment was carried out in fourteen clinics, out of sixty-one clinics in the selected districts to assess the compliance of AFHCs.

The checklist was developed by adapting the World Health Organization's (WHO) Global standards for quality healthcare services for adolescents to align with the Indian context[14] and using RKSK benchmarks.[13] The adaptation was carried through a Delphi process[19] involving panel of four adolescent health experts. Throughout the process, several items were excluded from the checklist, such as vaccine availability, ophthalmoscope, injectable

For peer review only

contraceptives, refrigerator, contraceptive implants, etc. These exclusions were guided by the India's Adolescent Health Strategy (2014), which outlines the essential requisites for AFHCs.[6] The trained study team also completed the Good Clinical Practice course offered by National Drug Abuse Treatment Clinical Trials Network (NDAT CTN).

The developed checklist comprised a total of 82 items (WHO standards=57 items; RKSK Benchmarks=25 items). Some of the items like Standard Operating Procedures, policy commitments, availability of condoms, contraceptives, etc. were observed and their responses were further confirmed through interaction with the concerned staff members [counsellors/ Auxillary Nurse Midwives (ANMs) / Medical Officers (MOs)] on the same day as per their convenient time without disturbing their day schedule. The embedded interactions approach was necessary, as specific items could be securely stored and concealed from direct observations. Table S1 provides the list of items verified by the interaction with the above-mentioned staff due to their involvement in handling adolescents and their health issues within AFHCs. The printed checklist was pre-tested by the study team in one AFHC within each study state, to assess its feasibility in the Indian context. Feedback received from the pre-testing primarily focused on the sequence of items, including potential deletion or addition. These inputs were incorporated into the final version of the checklist used for the main study. The two facilities used for the pre-testing phase were not included in the main study.

The observations were scheduled in consultation with the concerned authorities (counsellors/ ANMs/ MOs) at the facility, who were informed of the objective of conducting these assessments. Written informed consent was sought from the concerned staff of the health care facility before commencing the data collection. To avoid any potential bias in the responses, the checklist was not shared with the AFHC staff before data collection. The data collection process in each facility lasted for approximately 90-120 minutes.

For peer review only - http://bmjopen.bmj.com/site/about/guidelines.xhtml

The ethical clearance for the study was obtained from the Institutional Ethics Committee of the Public Health Foundation of India (Reference # TRC-IEC-342.1/17) along with approvals from the Indian Health Ministry's Screening Committee (2017-2250).

Measures

The RKSK operational framework[6,13] outlines seven benchmarks designed to ensure the provision of 'friendly' facility-based clinical and counselling services for adolescents at AFHC. Out of the seven benchmarks, only five benchmarks listed in Table 2 have been assessed in this study. The assessment of two benchmarks i.e. non-judgmental and competent health service providers, and awareness of the services to the community members was not feasible. Evaluating these benchmarks would have required exit interviews and observations of interaction between counsellors and adolescents, as well as interviews with community members. Unfortunately, these aspects were beyond the scope of our study, as conducting exit interviews with adolescents required parental consent. However, adolescents often visited the AFHCs either alone or with their peers. Similarly, observing the interaction of health worker and adolescent posed a threat to the confidentiality of the adolescents and could potentially impact their health seeking behaviour. The checklist included for assessing the RKSK benchmarks consisted of 25 items, detailed in Table 2, and Table S1.

Table 2. Variables assessing RKSK Benchmarks and International Standards

RKSK Benchmarks[6,13]	Number of items included*	Objective
Infrastructure- clean, bright and colourful	5	Provision of attractive, clean and comfortable health care facility to avail services.
Accessibility by adolescents (distance and convenient working hours)	3	Ensures easy access to health care facility through convenient working days/hours and local transportation.
Awareness about the clinic and range of service it provides (IEC, Proper Signage)	6	Provision of clearly visible sign board in regional language and information, education, and

For peer review only - http://bmjopen.bmj.com/site/about/guidelines.xhtml

		communication material to provide awareness about the presence of health facility and services available
Privacy and confidentiality	10	Ensures privacy in service provision through segregated areas for different services and maintain confidentiality by securing case records and registers under lock and key, accessible to only authorised AFHC staff
Referral from the periphery/community and further referral linkages with the higher facilities and specialty clinics	1	Provision of referral services to higher facilities to improve quality of healthcare among adolescents.
**Total Items**	25	
**WHO global standards for quality health care services for adolescents**[14]		
Standard 1- Adolescents' health literacy	3	To make adolescents knowledgeable about their own health and aware about where and when to receive health services.
Standard 2- Community Support**	0	To ensure that parents, guardians, other community members and community organizations recognize the value of providing health services to adolescents and support all adolescents to utilise the health services they need.
Standard 3- Appropriate package of services	2	Provision of package of services including information, counselling, diagnostic, treatment and care at the health facility to fulfil the needs of all adolescents.
Standard 4- Providers' competencies	10	Healthcare providers' attitudes, knowledge and skills are at the core of quality service provision. This standard ensures that health-care providers have the technical competence required to provide effective health services to adolescents. Adolescents' rights to information, privacy, confidentiality, non-discrimination, non-judgemental attitude and respect are protected and fulfilled by the healthcare providers

For peer review only - http://bmjopen.bmj.com/site/about/guidelines.xhtml

Standard 5- Facility characteristics	34	The health facility has convenient operating hours, a welcoming and clean environment and maintains privacy and confidentiality. It has the equipment, medicines, supplies and technology needed to ensure effective service provision to adolescents.
Standard 6- Equity and non-discrimination	4	Ensures provision of equitable care to all adolescents irrespective of their ability to pay, age, sex, marital status, education, ethnic origin, sexual orientation or other characteristics.
Standard 7- Data and quality improvement	3	The standard, stresses on the importance of the facility's actions to collect, analyse and use data on cause-specific service utilization and quality of care, disaggregated by age and sex to support quality improvement
Standard 8- Adolescents' participation	1	Ensures adolescents' participation in the planning, monitoring and evaluation of health services and in decisions regarding their own care. It also emphasizes on adolescents' participation in certain aspects of service provision.
Total	57	

*Details of the items are present in Table S1

** Standard 2 not assessed since exit interviews with adolescents were not conducted.

Similarly, to assess compliance with international standards, seven out of eight global standards defined in the WHO global standards for quality healthcare services for adolescents[14] were included in the checklist (Table 2). Community support (Standard 2) was not assessed as exit interviews with adolescent clients were needed. The developed checklist (Table 2 and S2) included only 57 items covering the WHO standards relevant to the Indian context.[20]

Data analysis

Data were entered into EpiInfo[21] and extracted into Excel for coding, cleaning, and analysis. For assessing compliance with international standards, a score was created for each standard as described in the WHO scoring guidelines.[22] Each item in the checklist was dichotomous

For peer review only - http://bmjopen.bmj.com/site/about/guidelines.xhtml

(yes/no) with “Yes” awarded 1 point, and “No” awarded 0 point. A summation of all the questions in each standard was used to obtain the total score for that standard. The maximum score was obtained by adding the number of items in that standard. The percentage score (%) for each standard was calculated by taking the score, scored by the AFHC, dividing it by the maximum score for the standard, and then multiplying by 100 for the standard. Percentage scores (%) were classified as: 1) ≤10%: not meeting standard, 2) 10-40%: needs major improvement, 3) 40-80%: needs some improvement, and 4) ≥80% or more: meets the standard.[20] Since, no scoring has been developed for assessing compliance to the RKSK benchmarks, WHO scoring guidelines[22] were adopted for assessing compliance of AFHCs with RKSK benchmarks.

Results

Out of the total fourteen clinics included, eight were from Maharashtra (Nashik: 4 and Yavatmal: 4), while six were from Madhya Pradesh (Panna: 3 and Damoh: 3). Two clinics from Maharashtra were additional, due to the presence of AFHCs at additional level of the health system i.e. sub-district hospital. Table 3 describes the characteristics of the 14 clinics including functional AFHC days and their operating hours. The maximum duration during which adolescents could avail services in a day was seven hours, while the minimum duration was four hours.

Table 3: Descriptive characteristics of AFHCs (n=14)

Clinic ID	AFHC Location at Health System	State	Number of operational days in week	Total Operational Hours
1	Government Medical College	Maharashtra	2	6
2	District Hospital	Maharashtra	5	7
3	District Hospital	Madhya Pradesh	6	7

For peer review only - http://bmjopen.bmj.com/site/about/guidelines.xhtml

4	District Hospital	Madhya Pradesh	6	7
5	Sub District Hospital	Maharashtra	6	7
6	Sub District Hospital	Maharashtra	6	4
7	Rural Hospital	Maharashtra	5	4
8	Rural Hospital	Maharashtra	6	2
9	Community Health Centre	Madhya Pradesh	6	7
10	Community Health Centre	Madhya Pradesh	6	7
11	Primary Health Centre	Maharashtra	1	4
12	Primary Health Centre	Maharashtra	1	4
[13]	Primary Health Centre	Madhya Pradesh	1	4
14	Primary Health Centre	Madhya Pradesh	1	4

The following section elucidates the compliance of the AFHCs where the assessment was undertaken with national RKSK benchmarks and WHO global standards of quality health services for adolescents.

RKSK Benchmarks

Infrastructure- clean, bright and colourful

Out of 14 AFHCs, half of the AFHCs required only some improvement (percentage score 40%-80%) in providing clean, bright and colourful infrastructure, while 5 AFHCs (35.7%) met this benchmark as their score was ≥80%. One AFHC (7.1%) required major improvements as the score was between 10%-40% and the other (7.1%) lacked compliance as the percentage score was 0 (Table 4). The item-wise analysis (Table S1) spotlights that as per the observations, majority of the clinics (n=11, 78.5%) had clean surroundings and more than half had adequate lighting in the waiting area (n=9, 64.3%), and clean waiting areas (n=8, 57.1%). A significant attention is required regarding toilet cleanliness (n=9, 64.3%) and the wall paints, furniture (n=8, 57.1%).

For peer review only - http://bmjopen.bmj.com/site/about/guidelines.xhtml

Accessibility by the adolescents (distance and convenient working hours)

Table 4 showed majority of the AFHCs (n=10, 71.4%) had a percentage score ≥80% thereby meeting the benchmark of being accessible concerning distance and working hours to adolescents. As observed, all the 14 AFHCs were located at a place that was easily accessible via local transport such as buses and auto-rickshaws. Based on observations and interactions with AFHC staff, it showed that in the majority of AFHCs (n=11) the timing and functional days were as per the RKSK operational guidelines (Table S1). None of the AFHCs showed non-compliance (percentage score ≤10%) in terms of accessibility (Table 4).

Awareness about the clinic and range of services offered (IEC, Proper Signage)

Regarding generating awareness about the clinic using signage and the IEC materials, it was seen that three AFHCs (21.3%) achieved a perfect score of 100% while only one AFHC failed to meet the benchmark, with a percentage score below 10%. Of the remaining AFHCs, five (35.7%) needed major improvement as the percentage score was between 10%-40%, and the other five AFHCs (35.7%) required some improvement, having a percentage score between 40%-80% (Table 4). Item-wise analysis (Table S1) revealed that a significant number of AFHCs (n=10; 71.4%) had external signboards designating them as AFHC. Visibility and inclusion of operating hours were observed in eight clinics (57.1%). The item which lacked in more than half of the AFHCs was the availability of IEC material in regional language (n=9, 64.3%) and education material in the waiting area (n=8; 57.1%)

Privacy and confidentiality

Overall, the majority of the AFHCs (n=13, 92.9%) required only some improvements in maintaining the privacy and confidentiality of adolescents as the percentage score was between

For peer review only - http://bmjopen.bmj.com/site/about/guidelines.xhtml

Table 4: Performance of AFHC as per RKSK Benchmarks				
	Score Percentage			
**RKSK Benchmarks**	**≤10% (not meeting the benchmark)**	**10%-40% (needs major improvement)**	**40%-80% (need some improvement)**	**≥80% (met the benchmark)**

40%-80%. Only a single AFHC managed to fulfil this benchmark, achieving a percentage score ≥80% (Table 4). The item-wise analysis (Table S1) showed that the majority of clinics (n=10, 71.4%) were physically separated from the general OPD within the healthcare facility. Almost all AFHCs (n=13, 92.9%) maintained adolescent case records in a secure place, accessible only to authorized personnel i.e. counsellor or medical officer. This was observed and verified through interactions. Additionally, in 78.5% of AFHCs (n=11), these records and registers were kept under lock and key either by the counsellor or medical officer.

Observations corroborated by interactions also revealed that during the registration process, the confidentiality of adolescents' identity was maintained. Additionally, no one could see the adolescent client at the time of counselling due to a separate designated area (n=9, 64.3%). However, an area of concern pertains to the provision of separate areas for clinical services (n=10, 71.4%) and the disbursement of commodities (n=9; 64.3%) within the AFHCs (Table S1).

Referral from the periphery/community and further referral linkages with the higher facilities and specialty clinics

Only one AFHC attained a perfect percentage score of 100% signifying the successful accomplishment of the benchmark. While, the remaining AFHCs (92.9%) did not meet the benchmark, as ascertained through observations and verified by interactions. The item-wise analysis showed that only one AFHC had the necessary referral guidelines visibly displayed.

For peer review only - http://bmjopen.bmj.com/site/about/guidelines.xhtml

	N (%)			
Infrastructure- clean, bright and colorful (Max. Score 5)	1 (7.1)	1 (7.1)	7 (50)	5 (35.7)
Accessibility by the adolescents (distance and convenient working hours) (Max. Score 3)	0 (0)	2 (14.3)	2 (14.3)	10 (71.4)
Awareness about the clinic and range of service it provides (IEC, Proper Signage) (Max. Score 6)	1 (7.1)	5 (35.7)	5 (35.7)	3 (21.3)
Privacy and confidentiality (Max. Score 10)	0 (0)	0 (0)	13 (92.9)	1 (7.1)
Referral from the periphery/community and further referral linkages with the higher facilities and specialty clinics (Max. score 1)	13 (92.9)	0 (0)	0 (0)	1 (7.1)
N: Number of AFHC<br>% : Percentage of AFHC				

For peer review only

WHO global standard of quality health services for adolescents

Standard 1: Adolescents' health literacy

Only three AFHCs (21.4%) met this standard with 100% compliance (Table 5), Conversely, an equal number of AFHCs (n=3, 21.4%) lacked compliance, achieving a percentage score lower than 10%. The remaining AFHCs required major (n=3, 21.4%) or some modifications (n= 5, 35.7%) was evident, as their score fall between 10%-40% and 40%-80%, respectively (Table 5). The item-wise analysis highlighted in Table S2 that while a majority of AFHCs (N=8; 57.1%) displayed visible signboards indicating their operating hours, more than half of AFHCs (n=8; 57.1%) lacked resources specifically targeting adolescents for information, education and communication in their waiting areas.

Standard 3: Appropriate package of services

Only one AFHC (7.1%) achieved a percentage score of 50%, indicating the need for some improvement due to the score falling between 40%-80% (Table 5). Conversely, the remaining AFHCs (n=13, 92.9%) fell short of meeting the standards, given their percentage scores were less than 10%. The item-wise analysis (Table S2) highlighted that both reference guidelines (n=13, 92.9%) and Standard Operating Procedures (SOPs) (n=14, 100%) for which services to be offered within the facility and community were not visibly displayed. This was corroborated through interactions.

Standard 4: Providers' competencies

Four AFHCs (28.6%) achieved percentage scores ranged from 10% to 40%, while the remaining ten AFHCs (71.4%) fell short of meeting the standard, receiving percentage scores below 10% (Table 5). The item-wise analysis (Table S2) revealed that low scoring by AFHCs could be attributed to various factors. The absence of well-defined job descriptions for health

For peer review only - http://bmjopen.bmj.com/site/about/guidelines.xhtml

care providers such as counsellors (n=13, 92.9%), ANMs (n=14, 100%), specialists (n=14, 100%), nurses (n=14, 100%) and medical officers (n=13, 92.9%). In addition, the lack of policies addressing aspects like confidentiality and privacy (n=14, 100%), the provision of free or affordable services in all AFHCs (n=14, 100%) and a policy commitment to provide health service to all adolescents without discrimination (n=13, 92.9%) can also be contributing factor to the low scores. The availability of BMI growth charts for adolescents as a supportive tool for providing quality clinical services to adolescents was noted in only three AFHCs (21.5%).

Standard 5: Facility characteristics

Out of the 14 AFHCs evaluated, two (14.3%) demonstrated a need for some improvement, as their percentage scores ranged from 40% and 80%. The remaining AFHCs (n=12, 85.7%) needed significant improvements, given their percentage scores were between 10%-40% in order to meet the established standard for facility characteristics (Table 5). Item-wise analysis (Table S2) spotlighted the reasons for the lack of compliance. This included lack of drinking water facility in the waiting area, the non-display of SOPs/ guidelines pertaining to the privacy and confidentiality of adolescents, SOPs outlining staff responsibilities, SOPs for minimizing waiting times, SOPs on how to provide services to adolescents, with or without an appointment. Several other concerns noted in more than 80% of AFHCs were the absence of provision for safe waste disposal, disposal of sharp objects, and the availability of essential equipment like measuring tape, stadiometer, contraceptive pills (oral and emergency), pregnancy kits and blood pressure measurement instruments etc.

Standard 6: Equity and non-discrimination

The majority of the AFHCs (n=13, 92.9%) failed to meet the established standard for equity and non-discrimination (Table 5). Only one AFHC exhibited a limited degree of compliance, attaining a percentage score of 25%, which falls within the range of 10%-40%. Table S2

For peer review only - http://bmjopen.bmj.com/site/about/guidelines.xhtml

showed that a significant number of AFHCs (n=13, 92.9%) did not display a policy commitment to provide health services to all adolescents without any discrimination. Furthermore, other policies, guidelines and procedures related to the provision of free and affordable services, along with equitable services irrespective of sex, marital status, and age were lacking across all clinics, as both observed and verified through interactions (Table S2).

Standard 7: Data and quality improvement

The majority of the AFHCs (n=10, 71.4%) exhibited a need for improvement, as their percentage scores ranged between 40%-80% while two AFHCs (14.3%) achieved a perfect 100% score (Table 5). Table S2, a detailed item-wise analysis showed that all the observed AFHCs (n=14) maintained an enrolment register, and a majority of AFHCs (n=12, 85.7%) possessed counselling registers. However, the stock register was not kept separate for AFHC (n=12, 85.7%)

Standard 8: Adolescents' participation

The assessment of adolescent participation involved inspection of the display of informed consent guidelines or SOPs at the facilities. Unfortunately, none of the fourteen observed AFHCs had any such guidelines or SOPs exhibited, resulting in their failure to adhere to the WHO standard for quality healthcare services for adolescents (Table 5).

Table 5: AFHC Performance as per the adapted WHO global standards for quality health care services for adolescents[14]

**WHO standards**	**Percentage Score**			
	**N (%)**			
	**≤ 10% (not meeting the standard)**	**10-40% (needs major improvement)**	**40-80% (needs some improvement)**	**≥ 80% (met the standard)**
**Standard 1- Adolescents' health literacy**	3 (21.4)	3 (21.4)	5 (35.7)	3 (21.4)

For peer review only

(Max. Score 3)				
**Standard 3- Appropriate package of services** (Max. Score 2)	13 (92.9)	0 (0)	1 (7.1)	0 (0)
**Standard 4- Providers' competencies** (Max Score 10)	10 (71.4)	4 (28.6)	0 (0)	0 (0)
**Standard 5- Facility characteristics** (Max. Score 34)	0 (0)	12 (85.7)	2 (14.3)	0 (0)
**Standard 6- Equity and non-discrimination** (Max. Score 4)	13 (92.9)	1 (7.1)	0 (0)	0 (0)
**Standard 7- Data and quality improvement** (Max Score 3)	0 (0)	2 (14.3)	10 (71.4)	2 (14.3)
**Standard 8- Adolescents' participation** (Max Score 1)	14 (100)	0 (0)	0 (0)	0 (0)

Discussion

To the best of our knowledge, this is the first study conducted in India that examines the compliance of AFHCs with both national benchmarks and global standards for quality care. Evidence from previous studies conducted in India have primarily evaluated AFHC compliance based on WHO standards, Indian Public Health Standards, or checklists from the National Health Mission.[23-26] However, adopting a global standard approach further enables critical evaluation of various facets of the quality of AFHS offered at a global level. It would not only

For peer review only - http://bmjopen.bmj.com/site/about/guidelines.xhtml

help minimize variations in service quality but also assist in comparing results across different countries.

RKSK Benchmarks

Our study revealed that AFHCs were compliant with the national benchmarks, indicating the government's commitment to holistic adolescent health, by setting up AFHCs under RKSK. Successful compliance with RKSK benchmarks was indicated by a host of reasons, including awareness of the AFHC through signage, convenient working hours, functional days and clean surroundings. Similar findings have been observed in the state of Ahmedabad[24] and West Bengal.[27] Moreover, AFHCs in both the study states were accessible by local transport, unlike AFHCs in Rajasthan, where ease of accessibility was a major concern.[28] Study results showed that the privacy and confidentiality of the patient were maintained, as all case records were kept in secured place and only authorized personnel (counsellor, ANM and medical officer) had access to retrieve them. Other requisites pertaining to privacy and confidentiality in adolescent services, like 'the provision of dedicated space for adolescents in clinics' have been one of the pressing issues highlighted in the literature.[29,30] Similar to AFHCs in Rajasthan, Jharkhand and Maharashtra,[31] more than half of the AFHCs included in our study, struggled to provide a separate waiting room and a separate area for clinical services and commodity disbursement for adolescents, while contradicting other regional AFHCs in Chandigarh and Kolkata.[32] The lack of these separate areas can threaten adolescent confidentiality and can act as a barrier in seeking and receiving appropriate medical services. Evidence highlights that adolescents who are assured of confidentiality have a greater willingness to discuss sensitive information like substance use, mental health, and sexual history.[33,34] Recently, MOHFW released guidelines to set up a model AFHC in each district hospital, where all the doors and windows of the clinic must have curtains.[35] To address this gap, the low-cost approach can be scaled up in all AFHCs irrespective of the level of the health

For peer review only - http://bmjopen.bmj.com/site/about/guidelines.xhtml

system. This involves separating areas for clinical services, counselling, and commodity disbursement using curtains.

These AFHCs are seen as crucial 'information hubs' that cater to a diverse range of adolescent health needs. IEC is a powerful tool to bring about social behaviour change and promote a culture of 'health-seeking behaviour' among adolescents, with an emphasis on health promotion and prevention.[36] Therefore, the absence of IEC materials, specifically in the regional language at the health facility, can act as a hurdle in utilization of the health services by adolescents. Studies across various Indian states including Gujarat (Ahmedabad),[24] Rajasthan[28] replicate the finding of limited IEC material at the AFHCs, while AFHCs in West Bengal [27] provided IEC material on Sexual and Reproductive Health (SRH). To make the Indian AFHC compliant to this benchmark, key is to have a combination of no-tech and high-tech resources in AFHCs that may assist in catering to a wider range of literacy levels of adolescents. No tech can include printed IEC materials such as posters, flipbooks,[36] and activity books and high tech can include animated movies, short bites on adolescent health issues in the clinic waiting area as seen in clinics of Sweden[37] and Ghana.[38]

Additional lacunae, highlighted as per the RKSK benchmark in our selected AFHC, included the lack of guidelines on services, such as referral guidelines, further limiting the utilization of quality services. These findings are consistent with other national[29] and international studies[39,40] where most AFHCs lack guidelines and SOPs'. These guidelines are a roadmap to a strong connection between various healthcare systems, ensuring efficient use of medical services. The introduction of referral guidelines enhances effective service delivery by reducing patient waiting time, overcrowding and improving patient in-and-out flow. It is also anticipated to raise the bar for patient management by appropriate use of investigations, patient counselling, and other patient-specific program services.[41] This emphasis the need for

For peer review only - http://bmjopen.bmj.com/site/about/guidelines.xhtml

referral guidelines for AFHC in India for creating an organized, efficient, and patient-centred healthcare system.

WHO global standard of quality health services for adolescents

Besides, the aforementioned strengths and limitations of AFHCs, identified using national benchmarks, the study also assessed the compliance of AFHCs to WHO global standards for adolescent-friendly health services. In addition, to some of the overlapping observations with the RKSK benchmarks, findings related to the WHO standards suggest a needed improvement in the observed AFHCs on clear and defined job descriptions, guidelines and SOPs.

Majority of the AFHCs included in the study lacked clearly defined job descriptions for medical officers, counsellors, nurses/ANMs etc and thus might contribute to ambiguity in the delivery of services. To support this finding, a recent assessment in Bhutan highlighted that more than half of healthcare providers (58%) did not provide SRH-related services to adolescents and 74% said their facilities did not have a guidelines or an SOP to provide equal and standard services to adolescents.[2] Similarly, a study in Ahmedabad (India) reveals shortcomings in the knowledge and technical capabilities of healthcare providers in areas related to health promotion, disease prevention, and management for adolescents.[24] This underscores the necessity of well-defined and precise job description for each health worker in AFHCs to ensure their effective functioning and and the delivery of comprehensive services to adolescents. Similarly, in AFHCs observed in two study states, the rights of adolescents to information, including privacy, confidentiality, non-discrimination, and non-judgemental attitude were not displayed, hindering the structural quality. Likewise, studies from India[28,31] and across the globe have reported judgemental and discriminatory behaviour,[42,43] and breach of confidentiality[42] by healthcare providers. Strong policies and protocols can enhance the quality of healthcare in India as evident in Ghana[38] where

For peer review only - http://bmjopen.bmj.com/site/about/guidelines.xhtml

adolescents' positive perception of quality healthcare was influenced by fair, non-discriminatory, respectful, competent and trustworthy healthcare providers. Compliance at the global level, therefore, emphasizes the need for organizing training programs such as those conducted in Tanzania[44] to improve the youth friendliness of the AFHCs and to ensure that these practices are consistently upheld protocols, guidelines, and SOPs in AFHCs. Displaying written statements can help alleviate fear and confusion about services and adolescent rights. Additionally, it also guarantees that health professionals recall their routine work, thereby ensuring continuity of all services rendered meet quality standards, thereby improving adolescent satisfaction.[45]

Moreover, while most of the recruited AFHCs had clean surroundings and proper signage; the absence of other infrastructural characteristics in the two AFHCs such as clean drinking water, safe storage, appropriate disposal of waste, functioning equipment and medical supplies need maintenance for effective healthcare delivery. The need for infrastructural and medical supplies for effective and appropriate service delivery and adolescent satisfaction has been substantiated in the assessment of services across countries.[38,46] The finding of insufficient medical supplies and equipment is congruent with facilities in Rajasthan (India)[28] and Uganda,[40] Nigeria[47] while contrast to the AFHC in Dehana district, Ethiopia[39] where availability of facility characteristics was found to be appropriate. Further, to support quality improvement adolescent participation holds prime importance, as healthcare services should be participant-sensitive and reflect their needs.

The study findings also showed that the unavailability of guidelines on informed consent to assess adolescent participation, is similar to facilities in the East and Southern Africa Region including[38] and those in Comoros, Mozambique, Zambia, etc., suggesting advocating for the development of youth-responsive health systems rather than just providing health care that is friendly to young people.[48]

For peer review only - http://bmjopen.bmj.com/site/about/guidelines.xhtml

Going forward, fulfilling certain aspects of national benchmarks is indicative of the Government of India's commitment to strengthening adolescent health. Nevertheless, there is a need to strengthen the existing AFHC as the lack of access to youth-friendly services paves the way for risky health behaviours among adolescents and exposes them to numerous vulnerabilities.[49] The AFHCs can be strengthened using the approach of community engagement and participatory co-design with youth at the centre to maximize access, uptake and coverage[50,51] by fostering ownership and assessing the impact of this approach in India. Furthermore, there is the potential for future research dedicating to establishing unified criteria for India by amalgamation the RKSK benchmarks with the global standards set by the WHO. This endeavour could utilize a co-creation approach, actively engaging service providers such as medical officers, ANMs and counsellors, as well as beneficiaries, particularly adolescents, in the process. This study provides data on the standard of adolescent-friendly health clinics in public health facilities in India. The findings should be interpreted with caution due to the limited geographical scope of the study. The sample included one clinic from each level of the health system in the selected study districts of Maharashtra and Madhya Pradesh and thus may not be generalizable to the entire country. In addition, exclusion of exit interviews with adolescent clients and community members did not permit assessment of all national benchmark and global standards. Inclusion of diverse methods like exit interviews with adolescent clients or employing mystery client methodology would have provided a comprehensive understanding of the healthcare worker's performance and the quality of care provided.

Conclusion

This study provides a window of opportunity for improvement by identifying few gaps in existing functional AFHCs. It is essential to address these gaps to create adolescent-friendly health facilities that can contribute to the survival, well-being and transformative growth of

For peer review only - http://bmjopen.bmj.com/site/about/guidelines.xhtml

adolescents in India. Taking cohesive actions by involving multiple stakeholders and using an approach of community engagement and participatory co-design is crucial to ensure that health services become acceptable, equitable, and accessible to all adolescents.

Patient and Public Involvement

Patients or the public were not involved in the design, conduct, reporting, or dissemination plans of our research.

Funding statement: This work was supported by Medical Research Council, UK. Grant number MC_PC_MR/P011446/1.

Competing Interests: None declared

Authors' Contribution: MA, SB and DB conceptualized this study. DB and HM were involved in data collection. DB, SB, HM, SK, AMJ and AM were involved in the analysis and interpretation of the findings. DB, SB, AM and HM drafted the manuscript. MA, AMJ and SD, critically reviewed the manuscript.

A data sharing statement: Data are available on reasonable request. As per our Institutional' s data sharing policy, prior approval is required from the Research Management Committee (RMC) and the Principal Investigator (PI) of the study. After approval of request from the Committee, de-identified data can only be shared upon request.

Acknowledgments: We would like to express our sincere gratitude to all the staff of the Adolescent Friendly Health Clinics in Maharashtra and Madhya Pradesh included in this study. We also thank Ms. Gayatri Nayak, Consultant (Public Health Foundation of India) to facilitate the data collection.

References

1. World Health Organisation. Making health services adolescent friendly: developing

For peer review only - http://bmjopen.bmj.com/site/about/guidelines.xhtml

national quality standards for adolescent friendly health services. 2012. Available: https://apps.who.int/iris/bitstream/handle/10665/75217/9789241503594_eng.pdf

2. World Health Organisation. Rapid assessment of the implementation of adolescent health programmes in countries of South-East Asia - Regional Summary. 2021. Available: https://apps.who.int/iris/bitstream/handle/10665/342587/9789290228639-eng.pdf?sequence=1&isAllowed=y

3. Kempers J, Ketting E, Lesco G. Cost analysis and exploratory cost-effectiveness of youth-friendly sexual and reproductive health services in the Republic of Moldova. *BMC Health Serv Res* 2014;**14**,316. doi: 10.1186/1472-6963-14-316

4. Ministry of Health and Family Welfare, Government of India. Adolescent Health (RKSK): National Health Mission. Available: https://www.nhm.gov.in/index1.php?lang=1&level=2&sublinkid=818&lid=221 [Accessed 24 Dec 2023]

5. Ministry of Health and Family Welfare, Government of India. Implementation Guidelines Rashtriya Kishor Sawasthya Karyakram(RKSK). 2018. Available: https://nhm.gov.in/New_Updates_2018/NHM_Components/RMNCHA/AH/guidelines/Implementation_Guidelines_Rashtriya_Kishor_Swasthya_Karyakram(RKSK)_2018.pdf

6. Adolescent Health Division. Ministry of Health and Family Welfare, Government of India. Operational framework- Translating strategy into programmes. 2014. Available: https://nhm.gov.in/images/pdf/programmes/RKSK/RKSK_Operational_Framework.pdf

7. Santhya K, Acharya R, Pandey N, *et al*. Understanding the Lives of Adolescents and Young Adults (UDAYA) in Bihar, India 2017. Available: https://www.projectudaya.in/wp-content/uploads/2018/08/UP-Report-pdf.pdf

For peer review only - http://bmjopen.bmj.com/site/about/guidelines.xhtml

8. Sowmya H, Singhe MS. Utilization of Adolescent Friendly Health Clinic (AFHC) services- A study of Dakshina Kannad district . *JAC: A J Compos Theory* 2020;**[13]**(10).

9. Mahalakshmy T, Premarajan KC, Soundappan K, *et al*. A mixed methods evaluation of adolescent friendly health clinic under national adolescent health program, Puducherry, India. *Indian J Pediatr* 2019;**86**(2):[13]2-[13]9. doi:10.1007/S12098-018-2755-4

10. Singh A, Joshi HS, Upadhyay D, *et al*. A study on awareness regarding treatment offered in adolescent-friendly health services in the rural area of Bareilly district. *Int J Adv Integr Med Sci* 2018;**3**(1):12-15. doi:10.5005/JP-JOURNALS-10050-101[13]

11. Wadhwa R, Chaudhary N, Bisht N, *et al*. Improving adolescent health services across high priority districts in 6 states of India: Learnings from an Integrated Reproductive Maternal Newborn Child and Adolescent Health Project. *Indian J Community Med* 2018;**43**(Suppl 1):S6. doi:10.4103/IJCM.IJCM_38_18

12. Ambresin AE, Bennett K, Patton GC, *et al*. Assessment of youth-friendly health care: a systematic review of indicators drawn from young people's perspectives. *J Adolesc Health* 20[13];**52**(6):670-681. doi:10.1016/j.jadohealth.2012.12.014

13. Ministry of Health & Family Welfare, Government of India. Adolescent Friendly Health Clinics (AFHCs): National Health Mission. Available: https://nhm.gov.in/index1.php?lang=1&level=3&sublinkid=1247&lid=421 [Accessed 19 July 2021].

14. World Health Organization, UNAIDS. Global standards for quality health-care services for adolescents: a guide to implement a standards-driven approach to improve the quality of health care services for adolescents. Volume 1: Standards and criteria.

For peer review only - http://bmjopen.bmj.com/site/about/guidelines.xhtml

World Health Organization. 2015. Available: https://apps.who.int/iris/bitstream/handle/10665/183935/9789241549332_vol1_eng.pdf?sequence=1&isAllowed=y

15. Centers for Disease Control and Prevention. US Department of Health and Human Services. Data Collection Methods for Program Evaluation: Observation. 2018. Available: https://www.cdc.gov/healthyyouth/evaluation/pdf/brief16.pdf

16. World Health Organization. Adolescent health. Available: https://www.who.int/health-topics/adolescent-health#tab=tab_1 [Accessed 2 Aug 2022]

17. Government of India Ministry of Home Affairs. Census of India | Office of the Registrar General & Census Commissioner, India. 2011. Available: https://censusindia.gov.in/census.website/ [Accessed 23 Aug 2022].

18. Arora M, Dringus S, Bahl D, *et al*. Engagement of health workers and peer educators from the National Adolescent Health Programme-Rashtriya Kishor Swasthya Karyakram during the COVID-19 pandemic: Findings from a situational analysis. *PLoS One* 2022;**17**(9 September):1-18. doi:10.[13]71/journal.pone.0266758

19. Chuenjitwongsa S. How to: conduct a delphi study. 2017. Available:https://www.cardiff.ac.uk/__data/assets/pdf_file/0010/1164961/how_to_conduct_a_delphistudy.pdf

20. World Health Organization, UNAIDS. Global standards for quality health-care services for adolescents: a guide to implement a standards-driven approach to improve the quality of health care services for adolescents. Volume 3: Tools to conduct quality and coverage measurement surveys to collect data about compliance with the global standards. World Health Organization. 2015. Available: https://apps.who.int/iris/bitstream/handle/10665/183935/9789241549332_vol3_eng.pdf?sequence=5&isAllowed=y

For peer review only - http://bmjopen.bmj.com/site/about/guidelines.xhtml

21. Centers for Disease Control and Prevention. Epi Info. Available: https://www.cdc.gov/epiinfo/index.html [Accessed 2 Aug 2022]

22. World Health Organization, UNAIDS. Global standards for quality health-care services for adolescents: a guide to implement a standards-driven approach to improve the quality of health care services for adolescents. Volume 4: Scoring sheets for data analysis. World Health Organization. 2015. Available: https://apps.who.int/iris/bitstream/handle/10665/183935/9789241549332_vol4_eng.pdf?sequence=6&isAllowed=y

23. Dolma Y. Rapid assessment of adolescent health clinic at sub-district hospital of Leh. *GJRA - Glob J Res Anal* 2018;**7**(2):141-142.

24. Dixit GT, Jain S, Mansuri F, *et al*. Adolescent friendly health services : where are we actually standing ? *Int J Community Med Public Heal* 2017;**4**(3):820-824.

25. Bhat AA, Jan Y, Jan R, *et al*. Adolescent friendly health centres: A review from North India. *IOSR Journal of Dental and Medical Sciences (IOSR-JDMS)* 2019;**18**(8):44-48. doi:10.9790/0853-1808044448

26. Bali S, Yadav K, Alok Y. A study of physical infrastructure and preparedness of Public Health Institution for providing adolescent friendly health services in Central India. *Indian J Prev Soc Med* 2020;**51**(4):195-203.

27. Biswas T, Majumdar KK. A study of the functioning of the adolescent friendly health clinics as well as the awareness and health seeking behaviour of adolescents attending the adolescent friendly health clinics in the Rampurhat health district, West Bengal, India. *Int J Community Med Public Health* 2022;**9**(11):4124-4[13]1. doi:10.18203/2394-6040.ijcmph20222907

28. Dayal R, Gundi M. Assessment of the quality of sexual and reproductive health services delivered to adolescents at Ujala clinics: A qualitative study in Rajasthan,

For peer review only - http://bmjopen.bmj.com/site/about/guidelines.xhtml

India. *PLoS One* 2022;**17**(1). doi:10.[13]71/JOURNAL.PONE.0261757

29. Awang H, Rahman AA, Sukeri S, *et al*. Making health services adolescent-friendly in Northeastern Peninsular Malaysia : A mixed-methods study. *Int J Environ Res Public Heal* 2020;**17**(4):[13]41. doi:10.3390/ijerph1704[13]41

30. Daley AM, Polifroni EC, Sadler LS. The essential elements of adolescent-friendly care in school-based health centers : A mixed methods study of the perspectives of nurse practitioners and adolescents. *J Pediatr Nurs* 2019;**47**:7-17. doi:10.1016/j.pedn.2019.03.005

31. Santhya KKG, Prakash R, Jejeebhoy SJ, et al. Accessing adolescent friendly health clinics in India: The perspectives of adolescents and youth. 2014. Available: https://www.popcouncil.org/uploads/pdfs/2014PGY_AFHC-IndiaReport.pdf

32. Yadav RJ, Mehta R, Pandey A, *et al*. Evaluation of adolescent-friendly health services in India. *Heal Popul Perspect Issues* 2009;**32**(2):96-104.

33. Ford CA, Thomsen SL, Compton B. Adolescents' interpretations of conditional confidentiality assurances. *J Adolesc Health*. 2001;29(3):156-159. doi:10.1016/s1054-[13]9x(01)00251-8.

34. Ford CA, Millstein SG, Halpern-Felsher BL, *et al*. Influence of physician confidentiality assurances on adolescents' willingness to disclose information and seek future health care. A randomized controlled trial. *JAMA*. 1997;278:1029–34. doi: 10.1001/jama.1997.03550120089044.

35. Ministry of Health and Family Welfare, Government of India. Guidelines for Operationalizing Model Adolescent Friendly Health Clinics (M-AFHC). 2022. Available: https://nhm.gov.in/images/pdf/programmes/RKSK/Guidelines_(M-%20AFHRCs).pdf

36. Sharma S, Akhtar F, Singh RK, *et al*. Validation of health education material for

For peer review only - http://bmjopen.bmj.com/site/about/guidelines.xhtml

youth: A step to ensure implementation fidelity in community-based interventions. *Healthcare (Basel)* 2019;**8(1)**:8. doi:10.3390/healthcare8010008.

37. Thomée S, Malm D, Christianson M, *et al*. Challenges and strategies for sustaining youth-friendly health services — a qualitative study from the perspective of professionals at youth clinics in northern Sweden. *Reprod Health* 2016;**[13]**(1):147. doi:10.1186/s12978-016-0261-6

38. Anaba, EA, Abuosi, AA. Assessing health care quality in adolescent clinics , implications for quality improvement. *Int J Heal Gov* 2019;(August 2018). doi:10.1108/IJHG-03-2018-0012

39. Gebrie M, Asrade G, Tsehay CT, *et al*. Quality of adolescent and youth-friendly health services in Dehana district public health facilities , northeast Ethiopia : Using the Donabedian quality framework. *PLoS One* 2021;**16**(10). doi:10.[13]71/journal.pone.0258718

40. Bukenya JN, Mulogo E, Kibira SPS, *et al*. Health facilities' readiness to provide friendly reproductive health services to young people aged 10-24 years in Wakiso district, Uganda. *Glob J Reprod Med* 2018;**2**(3). doi:10.19080/GJORM.2017.02.555588.Health

41. Department of Medical, Health and Family Welfare, Lucknow. Referral guidelines for health facilities in Uttar Pradesh. Available: http://dgmhup.gov.in/Documents/Referral_Guidelines.pdf

42. Geary RS, Gómez-olivé FX, Kahn K, *et al*. Barriers to and facilitators of the provision of a youth-friendly health services programme in rural South Africa. *BMC Health Serv Res* 2014:1-8. doi: 10.1186/1472-6963-14-259

43. Chilinda I, Hourahane G, Pindani M, *et al*. Attitude of health care providers towards adolescent sexual and reproductive health services in developing countries : A

For peer review only - http://bmjopen.bmj.com/site/about/guidelines.xhtml

Systematic Review. *Health (Irvine Calif)* 2014;(July):1706-17[13]. doi: 10.4236/health.2014.614203

44. Renju J, Andrew B, Nyalali K, *et al*. A process evaluation of the scale up of a youth-friendly health services initiative in northern Tanzania. *J Int AIDS Soc* 2010;**[13]**:32. doi:10.1186/1758-2652-[13]-32

45. Importance of SOPs Urban Primary Health and Wellness Centre**.** Available: https://vikaspedia.in/health/health-care-innovations/good--replicable-and-innovative-practices/importance-of-sops-urban-primary-health-and-wellness-centre. [Accessed 26 March 2023].

46. United Nations Population Fund, International Planned Parenthood Federation. Regional report: Assessment of adolescents and youth-friendly health service delivery in the East and Southern Africa region.2017. Available: https://esaro.unfpa.org/sites/default/files/pub-pdf/Assessment of Adolescents and Youth-Friendly Health Service Delivery in ESA.pdf

47. Osanyin Y. Report on assessment of facilities providing youth friendly health services in Nigeria. 20[13]. Available: http://www.ncceonline.edu.ng/download/files/Assessment of Youth Friendly Health Centers and Services.pdf

48. Sheikh K, Ranson MK, Gilson L. Explorations on people centredness in health systems. *Health Policy Plan* 2014;**29**:II1-II5. doi:10.1093/heapol/czu082

49. Partnership for Maternal, Newborn & Child Health. Ministry of Health and Family Welfare, Government of India. Centre staging health and well-being of adolescents and young people. 2023. Available: https://pmnch.who.int/docs/librariesprovider9/governance/g20-event-technical-session-1.pdf?sfvrsn=8f8ff09b_551.

For peer review only - http://bmjopen.bmj.com/site/about/guidelines.xhtml

50. United Nations Children's Fund (UNICEF). Adolescent development and participation.Available:https://www.unicef.org/india/what-we-do/adolescent-development-participation.[Accessed 19 Dec 2023].

51. Mackworth-Young C, Dringus S, Dauya E, *et al.* Putting youth at the centre: co-design of a community-based intervention to improve HIV outcomes among youth in Zimbabwe [version 2; peer review: 1 approved]. *Wellcome Open Res* 2022;**7**:53. https://doi.org/10.12688/wellcomeopenres.17531.2

For peer review only

Table S1: Compliance of Adolescent Friendly Health Clinics (AFHCs) with RKSK Benchmarks: Item-Wise Scores.

Items Under Each Benchmark	Method of assessment<br>Observation /<br>Observation and verified by interaction	AFHCs with Presence of item<br>N (%)
**Infrastructure- clean, bright and colorful (Max. Marks= 5)**		
Overall infrastructure of AFHC is bright with wall paints, color, furniture	Observation	6 (42.9)
Is the toilet clean?	Observation	5 (35.7)
Are the surroundings of the facility clean?	Observation	11 (78.5)
Does the waiting area have adequate lighting?	Observation	9 (64.3)
Does the waiting area seem clean overall?	Observation	8 (57.1)
**Can be easily accessed by the adolescents (distance and convenient working hours) (Max. Marks= 3)**		
Do the functional days of the AFHC match with the RKSK operational framework?	Observation and verified by interaction	11 (78.5)
Do the Operational Hours of the AFHC match with the RKSK operational framework?	Observation and verified by interaction	11 (78.5)
Is AFHC accessible to adolescents i.e. where adolescents can reach using the local transport?	Observation	14 (100)
**Awareness about the Clinic and its range of services (IEC, proper signage etc.) (Max. Marks= 6)**		
Signboard mentions that it is the AFHC	Observation	10 (71.4)
Signboard that mentions the facility's operating hours?	Observation	8 (57.1)
Signboard clearly visible?	Observation	8 (57.1)
Signboard displayed in the regional language?	Observation	7 (50)
Is the IEC material displayed in regional language?	Observation	5 (35.7)
Does the waiting area have information, education and communication materials specifically developed for adolescents?	Observation	6 (42.9)

For peer review only

**Maintains privacy and confidentiality (Max. Marks= 10)**		
Is the waiting area in a common area ?	Observation	8 (57.1)
AFHC is separated from general OPD	Observation	10 (71.4)
Separate area for Counselling services	Observation	9 (64.3)
Separate area for Clinical services	Observation	4 (28.6)
Separate area for commodities disbursement	Observation	5 (35.7)
There are curtains on the doors and windows	Observation	8 (57.1)
Case records are kept in a secure place, accessible only to authorized personnel	Observation and verified by interaction	13 (92.9)
The registers are kept under lock and key outside operating hours	Observation and verified by interaction	11 (78.5)
Information on the identity of the adolescent and the presenting issue are gathered in confidence during registration	Observation and verified by interaction	9 (64.3)
No one can see or hear an adolescent client from the outside during the consultation or counselling	Observation	9 (64.3)
**Referral from the periphery/community and further referral linkages with the higher facilities and specialty clinics. (Max. Marks= 1)**		
Referral guidelines	Observation and verified by interaction	1 (7.1)

For peer review only

Table S2: Compliance of Adolescent Friendly Health Clinics (AFHCs) with WHO Global standards for quality health-care services for adolescents: Item-Wise Scores.

Items Under Each Standard	Method of assessment<br>Observation<br>Observation and verified by interview	Yes
**Standard 1- Adolescents' health literacy (Max. Score= 3)**		
Signboard that mentions the facility's operating hours?	Observation	8 (57.1)
Signboard clearly visible?	Observation	8 (57.1)
Does the waiting area have information, education and communication materials specifically developed for adolescents?	Observation	6 (42.9)
**Standard 3- Appropriate package of services (Max. Score= 2)**		
Guidelines/SOPs for which services should be provided in the facility and in the community	Observation and verified by interview	0 (0)
Referral guidelines	Observation and verified by interview	1 (7.1)
**Standard 4- Providers' competencies (Max. Score= 10)**		
BMI growth charts for adolescents	Observation	3 (21.5)
The rights of adolescents to information, non-judgmental attitude and respectful care	Observation and verified by interview	1 (7.1)
The policy commitment of the health facility to provide health services to all adolescents without discrimination	Observation and verified by interview	1 (7.1)
The policy on confidentiality and privacy	Observation and verified by interview	0 (0)
The policy and procedure to ensure free or affordable service provision for adolescents	Observation and verified by interview	0 (0)
Job Description- Medical Officer	Observation and verified by interview	1 (7.1)

For peer review only

Job Description- Staff Nurse	Observation and verified by interview	0 (0)
Job Description- ANM	Observation and verified by interview	0 (0)
Job Description- Counsellor	Observation and verified by interview	1 (7.1)
Job Description- Specialist	Observation and verified by interview	0 (0)
**Standard 5- Facility characteristics (Max. Score= 34)**		
Does the waiting area have information, education and communication materials specifically developed for adolescents?	Observation	6 (42.9)
Does the waiting area have drinking water?	Observation	0 (0)
Does the waiting area seem welcoming overall?	Observation	6 (42.9)
Does the waiting area seem clean overall?	Observation	8 (57.1)
Is the toilet clean?	Observation	5 (35.7)
Does the facility have permanent electricity during working hours?	Observation	9 (64.3)
Does the facility have safe waste disposal?	Observation	3 (21.5)
Does the facility have safe storage and disposal of sharps?	Observation	3 (21.5)
Does the facility have adequate hand hygiene facilities that are located in or adjacent to the office/examination room?	Observation	6 (42.9)
Does the toilet have functioning hand hygiene facilities?	Observation	6 (42.9)
Are the surroundings of the facility clean?	Observation	11 (78.5)
Blood pressure measurement machine	Observation	2 (14.3)
Stethoscope	Observation	3 (21.5)

For peer review only

Clinical thermometer	Observation	2 (14.3)
Weighing scale	Observation	10 (71.4)
Measuring tape	Observation	1 (7.1)
Height Meter/Stadiometer	Observation	2 (14.3)
Hemoglobin Testing	Observation	3 (21.5)
Provision of Condoms	Observation and verified by interview	6 (42.9)
Provision of Oral Contraceptive Pills	Observation and verified by interview	3 (21.5)
Provision of Emergency Contraceptive Pills	Observation and verified by interview	1 (7.1)
Provision of Pregnancy Testing Kits	Observation and verified by interview	2 (14.3)
Paracetamol	Observation and verified by interview	3 (21.5)
Guidelines/SOPs on protecting the privacy and confidentiality of adolescents	Observation and verified by interview	0 (0)
Guidelines/SOPs on including staff responsibilities for making the health facility welcoming, convenient and clean	Observation and verified by interview	0 (0)
SOPs on how to minimize waiting time	Observation and verified by interview	0 (0)
SOPs on how to provide services to adolescents with or without an appointment	Observation and verified by interview	0 (0)
There are curtains on the doors and windows	Observation	8 (57.1)
Communication between reception staff and visitors is private and cannot be overheard, including from the waiting room	Observation	2 (14.3)
Case records are kept in a secure place, accessible only to authorized personnel	Observation and verified by interview	13 (92.9)
The registers are kept under lock and key outside operating hours	Observation and verified by interview	11 (78.5)

For peer review only

Information on the identity of the adolescent and the presenting issue are gathered in confidence during registration	Observation	9 (64.3)
In the offices/examining rooms, there is a screen to separate the examination area from the consultation area	Observation	4 (28.6)
No one can see or hear an adolescent client from the outside during the consultation or counselling	Observation	9 (64.3)
**Standard 6- Equity and non-discrimination (Max. Score= 4)**		
The policy commitment of the health facility to provide health services to all adolescents without discrimination	Observation and verified by interview	1 (7.1)
The policy and procedure to ensure free or affordable service provision for adolescents	Observation and verified by interview	0 (0)
Guidelines/SOPs on applying policies for free, or affordable, service provision to adolescents	Observation and verified by interview	0 (0)
Guidelines/SOPs on equitable service provision to all adolescents irrespective of their ability to pay, age, sex, marital status or other characteristics	Observation and verified by interview	0 (0)
**Standard 7- Data and quality improvement (Max. Score= 3)**		
Enrolment Register	Observation and verified by interview	14 (100)
Counselling Register	Observation and verified by interview	12 (85.7)
Stock Register	Observation and verified by interview	2 (14.3)
**Standard 8- Adolescents' participation (Max. Score= 1)**		
Guidelines/SOPs on informed consent	Observation and verified by interview	0 (0)

For peer review only

BMJ Publishing Group Limited (BMJ) disclaims all liability and responsibility arising from any reliance placed on this supplemental material which has been supplied by the author(s)

SRQR Reporting checklist for qualitative study.

Instructions to authors

Complete this checklist by entering the page numbers from your manuscript where readers will find each of the items listed below. SRQR reporting guidelines: O'Brien BC, Harris IB, Beckman TJ, Reed DA, Cook DA. Standards for reporting qualitative research: a synthesis of recommendations. Acad Med. 2014;89(9):1245-1251.

		Reporting Item	Page Number
**Title**			
	#1	Concise description of the nature and topic of the study identifying the study as qualitative or indicating the approach (e.g. ethnography, grounded theory) or data collection methods (e.g. interview, focus group) is recommended	Page 1
**Abstract**			Abstract
	#2	Summary of the key elements of the study using the abstract format of the intended publication; typically includes background, purpose, methods, results and conclusions	Page 1
**Introduction**			
Problem formulation	#3	Description and signifcance of the problem / phenomenon studied: review of relevant theory and empirical work; problem statement	Introduction Page 3-5
Purpose or research question	#4	Purpose of the study and specific objectives or questions	Introduction Page 5
**Methods**			
Qualitative approach and research paradigm	#5	Qualitative approach (e.g. ethnography, grounded theory, case study, phenomenolgy, narrative research) and guiding theory if appropriate; identifying the research paradigm (e.g. postpositivist, constructivist / interpretivist) is also recommended; rationale. The rationale should briefly discuss the justification for choosing that theory, approach, method or technique rather than other options available; the assumptions and limitations implicit in those choices and how those choices influence study conclusions and transferability. As appropriate the rationale for several items might be discussed together.	Data collection, Page 7<br>Data analysis: Page 8, Para 1
Researcher characteristics and reflexivity	#6	Researchers' characteristics that may influence the research, including personal attributes, qualifications / experience, relationship with participants, assumptions and / or presuppositions; potential or actual interaction between researchers' characteristics and the research questions, approach, methods, results and / or transferability	Data collection, page 7
Context	#7	Setting / site and salient contextual factors; rationale	Study setting and Sample selection Page 5
Sampling strategy	#8	How and why research participants, documents, or events were selected; criteria for deciding when no further sampling was necessary (e.g. sampling saturation); rationale	Study setting and sample selection Page 7
Ethical issues pertaining to human subjects	#9	Documentation of approval by an appropriate ethics review board and participant consent, or explanation for lack thereof; other confidentiality and data security issues	Page 8, Para 2 and Para 3
Data collection methods	#10	Types of data collected; details of data collection procedures including (as appropriate) start and stop dates of data collection and analysis, iterative process, triangulation of sources / methods, and modification of procedures in response to evolving study findings; rationale	Data Collection Page 7, 8

For peer review only

Hennegan J, *et al. BMJ Global Health* 2020; 5:e003433. doi: 10.1136/bmjgh-2020-003433

Data collection instruments and technologies	#11	Description of instruments (e.g. interview guides, questionnaires) and devices (e.g. audio recorders) used for data collection; if / how the instruments(s) changed over the course of the study	Data collection and procedure: Page 7
Units of study	#12	Number and relevant characteristics of participants, documents, or events included in the study; level of participation (could be reported in results)	Results: Page 13, 14
Data processing	#13	Methods for processing data prior to and during analysis, including transcription, data entry, data management and security, verification of data integrity, data coding, and anonymisation / deidentification of excerpts	Measures, Page 8, Data analysis: Page 12
Data analysis	#14	Process by which inferences, themes, etc. were identified and developed, including the researchers involved in data analysis; usually references a specific paradigm or approach; rationale	Data analysis: Page 12
Techniques to enhance trustworthiness	#15	Techniques to enhance trustworthiness and credibility of data analysis (e.g. member checking, audit trail, triangulation); rationale	Data analysis: Page 12
**Results/findings**			
Syntheses and interpretation	#16	Main findings (e.g. interpretations, inferences, and themes); might include development of a theory or model, or integration with prior research or theory	Results: Page 14-20
Links to empirical data	#17	Evidence (e.g. quotes, field notes, text excerpts, photographs) to substantiate analytic findings	NA
**Discussion**			
Intergration with prior work, implications, transferability and contribution(s) to the field	#18	Short summary of main findings; explanation of how findings and conclusions connect to, support, elaborate on, or challenge conclusions of earlier scholarship; discussion of scope of application / generalizability; identification of unique contributions(s) to scholarship in a discipline or field	Discussion: Page 21-25
Limitations	#19	Trustworthiness and limitations of findings	Discussion: Page 25
**Other**			
Conflicts of interest	#20	Potential sources of influence of perceived influence on study conduct and conclusions; how these were managed	Page 25
Funding	#21	Sources of funding and other support; role of funders in data collection, interpretation and reporting	Page 25

None The SRQR checklist is distributed with permission of Wolters Kluwer © 2014 by the Association of American Medical Colleges. This checklist can be completed online using https://www.goodreports.org/, a tool made by the EQUATOR Network in collaboration with Penelope.ai

For peer review only

Hennegan J, et al. BMJ Global Health 2020; 5:e003433. doi: 10.1136/bmjgh-2020-003433
